# Skin melanoma cells produce diverse gelsolin (GSN) isoforms, which play non-redundant roles in cells’ proliferation and motility

**DOI:** 10.1186/s12935-025-03876-x

**Published:** 2025-07-01

**Authors:** Ewa Mazurkiewicz-Stanek, Aleksandra Makowiecka, Iryna Kopernyk, Michał Majkowski, Anna Boguszewska-Czubara, Tomasz Trombik, Paweł Karpiński, Piotr Donizy, Antonina J. Mazur

**Affiliations:** 1 Department of Cell Pathology, Faculty of Biotechnology, University of Wroclaw, Wrocław, Poland; 2 Faculty of Biotechnology, University of Wroclaw, Wrocław, Poland; 3 Department of Biochemistry and Molecular Biology, Medical University of Lublin, Lublin, Poland; 4 Department of Genetics, Wroclaw Medical University, Wrocław, Poland; 5 Department of Clinical and Experimental Pathology, Wroclaw Medical University, Wrocław, Poland

**Keywords:** Gelsolin (GSN), Isoforms, Skin melanoma, Motility, Adhesion, Cancer, Phenotypic heterogeneity

## Abstract

**Background:**

Skin melanoma is a malignant tumor that becomes increasingly difficult to treat when diagnosed late. Previously, we demonstrated that high levels of gelsolin (GSN) correlate with the advanced stages of cutaneous melanoma. GSN can be produced as various isoforms due to alternative splicing and differing start codon positions. To date, no studies have been conducted to determine whether GSN diverse isoforms are produced by melanoma cells in vivo and melanoma cell lines. Therefore, nothing is known about the role of specific GSN isoforms in skin melanoma biology.

**Methods:**

We applied immunocytochemical staining to melanoma tissue samples to analyze the localization of GSN production within tumor samples. Additionally, we utilized bioinformatics analysis of transcript levels coding for selected GSN isoforms in publicly available gene transcript databases. To investigate the role of these GSN isoforms, we used the melanoma A375 cell line with *GSN* knockout to restore the production of only one GSN isoform at a time in these cells. We evaluated the modified cells' ability to migrate, spread, and regulate actin polymerization. We also tested the cells growing on laminin 1, a significant component of the basement membrane, and the melanoma microenvironment.

**Results:**

We found that *GSN* expression produces three GSN isoforms in human melanoma cell lines: two cytosolic (B and C) and one secretory (A). Furthermore, we noted the presence of GSN both intracellularly and extracellularly in melanoma tumor samples, indicating that human melanoma cells produce diverse GSN isoforms in vivo. We discovered that cells producing GSN-A invade more efficiently, while cells producing GSN-C form the longest filipodia and migrate the best in 2D conditions. Both GSN-B and -C decrease the amount of filamentous actin. On the other hand, cells producing GSN-A and –B exhibit a lower proliferation rate. Finally, we observed that tumors formed by the clones expressing individual GSN isoforms do not grow in zebrafish embryos.

**Conclusions:**

Overall, we demonstrate that GSN isoforms are produced as a mixture in melanoma cells and are not redundant in their function. Therefore, to support the well-being of melanoma cells, a mixture of GSN isoforms must be produced.

**Supplementary Information:**

The online version contains supplementary material available at 10.1186/s12935-025-03876-x.

## Background

Animal cells can change shape to adapt to the extracellular environment, move, undergo cell division, and facilitate exocytosis and endocytosis. The underlying mechanism for these processes is the continuous reorganization of the cytoskeleton. Major classes of cytoskeletal components include microtubules, actin filaments, intermediate filaments, and septins [1, 2]. The basic building block of the actin cytoskeleton is an actin monomer (globular actin, G-actin), which, in a reaction reliant on the hydrolysis of adenosine triphosphate (ATP) and magnesium ions, creates a polarized homopolymer structure known as a microfilament or actin filament (filamentous actin, F-actin) [1]. Variously organized actin filaments constitute the building blocks of F-actin-rich structures—lamellipodium, filopodium, stress fibers, adhesion foci, actin cortex, and podosome/invadopodium [1]. Crucial for cell migration, the orchestration of the dynamic reorganization of these structures depends on hundreds of actin-binding proteins (ABPs), whose activation relies on external and internal stimuli.

The gelsolin superfamily includes many proteins involved in regulating actin polymerization, such as vilin, adseverin, capG, advilin, supervilin, protovilin, fragmin, flightless protein I, and gelsolin (GSN) [3]. GSN (GSN), a 782-amino acid protein (isoform B), consists of six domains labeled G1-G6 [4]. It functions as an ABP capable of interacting with calcium ions and phosphoinositol 4,5-bisphosphate [PI(4,5)P_2_] [5]. There are at least three, at the different levels studied, isoforms of GSN: secretory, and two cytoplasmic (A, B, and C) [5]. All are encoded by a single gene but produced through alternative mRNA processing and transcription initiation at different sites [6, 7]. GSN-A, the secretory isoform, shares 93.6% of its amino acid sequence with GSN-B, except it has an additional 51 amino acid residues at the N-terminus. The first 27 amino acid residues in GSN-A's sequence form a signal peptide, which directs the newly synthesized peptide chain to the endoplasmic reticulum, where the signal peptide is removed. As a result, mature GSN-A is approximately 24 amino acid residues longer than GSN-B. In comparison, GSN-C includes 11 extra amino acids at its N-terminus relative to GSN-B [5]. Of the five cysteine (Cys) residues in the GSN-A and -B amino acid sequences, all occur as free thiols in the cytoplasmic isoform. In contrast, only three function as free thiols in isoform A. An additional disulfide bond between Cys188 and Cys201 stabilizes the G2 domain, indicating that intracellular GSN-B and extracellular GSN-A differ in their conformations. Conversely, GSN-C is characterized by the presence of two additional cysteine residues at the N-terminus, allowing for the formation of further intramolecular disulfide bonds in the protein [9]. Due to the absence of specific antibodies targeting individual isoforms of GSN, most studies concentrate on its general pool, occasionally distinguishing them only by their location, that is, secretory and intracellular GSN.

GSN is a multifunctional protein, and its most widely described role is its interaction with actin. In addition to contributing to the reorganization of the actin cytoskeleton, intracellular GSN has numerous other functions related to the regulation of cellular metabolism [3]. In the context of carcinogenesis, GSN plays a dual role. Reduced GSN expression has been observed in various cancers, including breast, colon, stomach, bladder, prostate, kidney, ovarian, and oral cancer [10]. In contrast, cervical cancer is characterized by increased GSN production [11]. In studies of pancreatic and lung cancer, the precise role of GSN remains unclear due to evidence showing that GSN has a dual effect on tumor progression [12, 13]. Transfecting bladder cancer cells with a construct encoding GSN-B has been shown to reduce their ability to form colonies [14].

Available data highlight a crucial role for secretory GSN in various physiological and pathological processes [15]. Secretory GSN has been shown to inhibit the activity of DNGR-1, a dendritic cell-specific sensor of tissue damage, which enhances the cross-presentation of dead cell-associated antigens, including tumor antigens. Through this interaction, GSN disrupts tumor antigen presentation, allowing tumor cells to evade immune responses [16]. Studies on ovarian cancer indicate that secretory GSN-A functions as an inflammatory modulator, contributing to the chemoresistance of tumor cells by disrupting the antitumor activity of macrophages within the tumor environment [17]. Studies by Tsai and colleagues suggest that secretory GSN could serve as a marker for distant metastasis in patients with colorectal cancer [18]. Proteomics-based analyses of plasma samples from patients with primary and metastatic tumors have demonstrated elevated levels of GSN in those with metastases compared to those without. Additionally, colorectal cancer cells exhibiting increased migratory potential have been found to produce more secretory GSN than their counterparts with reduced migratory capacity [18]. The mechanism through which GSN-A influences the metastatic potential of cancer cells has not been determined. GSN-C is the least understood isoform of GSN, with only a few reports on its function. GSN-C is found under physiological conditions only in oligodendrocytes, lungs, and testis [7]. It may play a role in myelogenesis [7] and myelin remodeling [19].

There are only a few publications regarding the role of GSN in skin melanoma. One of our previous studies demonstrated that knocking down *GSN* expression with siRNA resulted in changes to the morphology of A375 cells and decreased their migratory potential [20]. However, these studies were limited to evaluating the actin polymerization state and cell migration through a porous filter toward a chemoattractant. Later, we demonstrated that melanoma cell lines produce GSN in significantly higher amounts compared to other types of cancer [21]. Additionally, we identified new proteins that interact with GSN in both the nuclear and cytoplasmic fractions [21]. More recently, we demonstrated that GSN-B interacts directly with ribosomal protein SA, likely recruiting ribosomes to the plasma membrane and regulating local translation [22]. Additionally, elevated levels of GSN and RPSA were linked to the progression of skin melanoma and a poorer prognosis for patients. Our other study indicated that human cutaneous melanoma cells with a *GSN *knockout exhibit significantly lower invasive and migratory potential compared to control cells [23]. Therefore, now we aimed to investigate the function of individual GSN isoforms (A, B, C) in human skin melanoma cells regarding their motility and adhesion, as this aspect has not been examined previously.

## Results

### Human melanoma cells express different isoforms of GSN

**Fig. 1:**
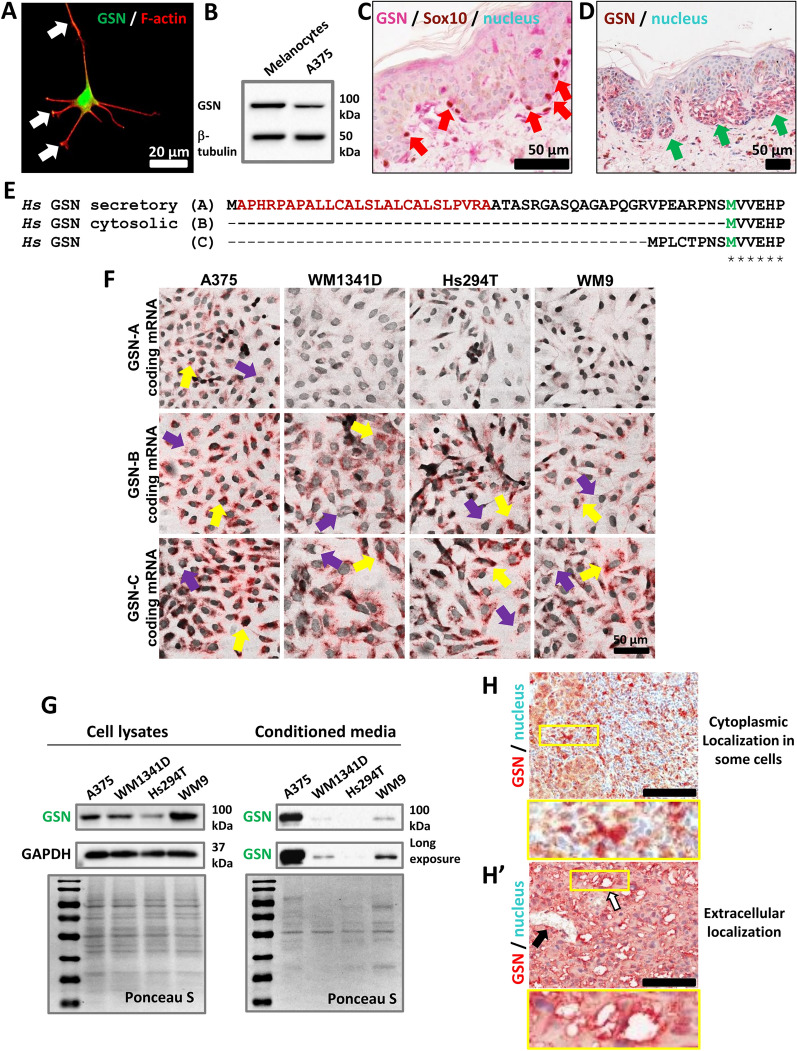
Human cutaneous melanocytes and melanoma cells produce GSN at high levels and various isoforms. **A** Detection of GSN and F-actin in cultured human skin melanocytes via immunofluorescent staining. White arrows point at protrusions. **B** Western blot analysis of lysates from human skin melanocytes and cutaneous melanoma cells—A375 cells—using antibodies directed against GSN and β-tubulin as a loading control. 20 μg of protein was loaded into each well. **C** IHC staining of healthy human skin tissue to detect GSN and Sox10, a melanocyte marker. Red arrows indicate positive cells for GSN and Sox10 located on the basement membrane. **D** IHC analysis of skin melanoma in situ without dermal invasion shows enhanced GSN immunoreactivity in neoplastic cells with high GSN levels in the extracellular (stromal) compartment of the dermis (green arrows). **E** Alignment of N-termini of human GSN isoforms analyzed in the study, i.e., A, B, and C. “asterisk”—no substitutions; “dot”—semi-conserved substitution. The signal peptide of the secretory isoform is highlighted in red, while M, shown in green, indicates the start of the GSN-B isoform. CLUSTAL format alignment was conducted using MAFFT (v7.511) [24, 25]. **F** BaseScope™ analysis of four melanoma cell lines to detect in situ transcripts coding for GSN-A, GSN-B, and GSN-C in human skin melanoma cells. Yellow and violet arrows highlight cells with prominent or low detected signals. **G** Western blot analysis of lysates from human skin melanocytes and cutaneous melanoma cells—A375 cells—using antibodies directed against GSN and β-tubulin as a loading control. Western blot analysis of conditioned media collected from human cutaneous melanoma cell cultures. The membrane was stained with Ponceau S as a loading control. 20 μg of protein was loaded into each well. **H–H’** IHC staining of human skin melanoma tissues to detect GSN (red signal). Samples were counterstained with hematoxylin to visualize the cell nucleus (blue). Black and white arrows indicate vascular spaces and probable vasculogenic mimicry, respectively, where there is a high GSN signal at the rim. **H–H’** samples from two different patients. Boxed areas are shown at higher magnification

First, to examine whether normal cutaneous melanocytes produce GSN, we stained cultured human skin melanocytes to detect GSN and observed a high amount of GSN localized in the cell body of melanocytes (Fig. 1A). Additionally, it was present at the tips of melanocytic protrusions. Furthermore, we prepared lysates of skin melanocytes and noted that GSN was produced at a high level compared to the human melanoma cell line (Fig. 1B). Moreover, we stained a fragment of healthy human skin with antibodies directed against GSN and Sox10 to detect melanocytes. GSN is produced in large amounts by melanocytes residing on the basement membrane (Fig. 1C). Then, we checked whether GSN is produced in melanoma tissue in patients. Indeed, as shown for melanoma, in situ tumor cells exhibited high levels of GSN (Fig. 1D).

GSN isoforms vary in their N-terminus (Fig. 1E; the full alignment of amino acid sequences is shown in Fig. S1). Unfortunately, no specific antibodies are available that recognize particular GSN isoforms. Therefore, we analyzed whether transcripts coding for GSN-A, -B, and -C exist in skin melanoma samples. Furthermore, we compared the levels of specific transcripts between normal skin and cancerous tissue. We noted that while the GSN-A transcript level is decreased in melanoma tissue, the levels of GSN-B and GSN-C are significantly elevated in tumor tissue compared to normal skin (Fig. S2). To determine if human skin melanoma cell lines express the studied isoforms of GSN, we performed the BaseScope™ procedure to detect in situ transcripts coding for GSN-A, -B, and -C in selected human cutaneous melanoma cell lines: A375, WM1341D, Hs294T, and WM9 (Fig. 1F). Interestingly, we found a high number of positive cells for the presence of the GSN-A-coding transcript in A375 cells, whereas other cell lines exhibited relatively low or no signal for this transcript type. In contrast, there were many positive cells in all tested cell lines for the presence of transcripts for GSN-B and -C. Notably, for the detection of transcripts coding for GSN-B or -C, we observed cells with varying amounts of this type of transcript. This mirrors our previous observation that melanoma cells produce various levels of GSN [23]. A similar observation was made for A375 cells concerning the GSN-A-coding transcript. Negative and positive controls for the BaseScope™ procedure are shown in Fig. S3. In the next step, we examined the level of GSN in cell lysates and conditioned medium collected from melanoma cell lines using Western blot analysis (Fig. 1G). GSN was detected in both cell lysates and conditioned medium collected from cultures of human cutaneous melanoma cells maintained in serum-free medium for 24 h. Due to differences in GSN production levels across different melanoma cell lines, it was necessary to extend the acquisition time of the chemiluminescent signal to detect GSN in cell lines with lower levels of this protein in the conditioned medium. Finally, we subjected skin melanoma samples from several patients to immunohistochemical (IHC) analysis to determine the localization of GSN in tumor samples. We observed both cellular and extracellular presence of GSN in various samples (Fig. 1H–H’), indicating that skin melanoma cells must produce at least one cytosolic isoform and one secretory isoform of GSN. Lastly, we examined whether, in patient samples, the localization of GSN is homogeneous or heterogeneous. We present cases showing that GSN can be localized intracellularly (Fig. 1H) and likely extracellularly near the lumen of extracellular spaces with disrupted cell–cell adhesion resembling vascular mimicry (Fig. 1H’). Melanoma tissue from the same patient (referring to Fig. 1H') stained with eosin and hematoxylin to visualize these structures is shown in Fig. S4.

Overall, GSN is present in melanocytes and melanoma tissue. Furthermore, GSN’s localization can be both intracellular and extracellular, and we demonstrate that several melanoma cell lines produce all three studied here GSN isoforms, resulting in the presence of both secreted and intracellular GSN. Lastly, we show that in melanoma tissue, the transcript for GSN-B is produced at the highest level among the transcripts coding for the studied GSN isoforms.

### Expression of individual GSN isoforms in A375 cells, which lack endogenous *GSN* expression

**Fig. 2:**
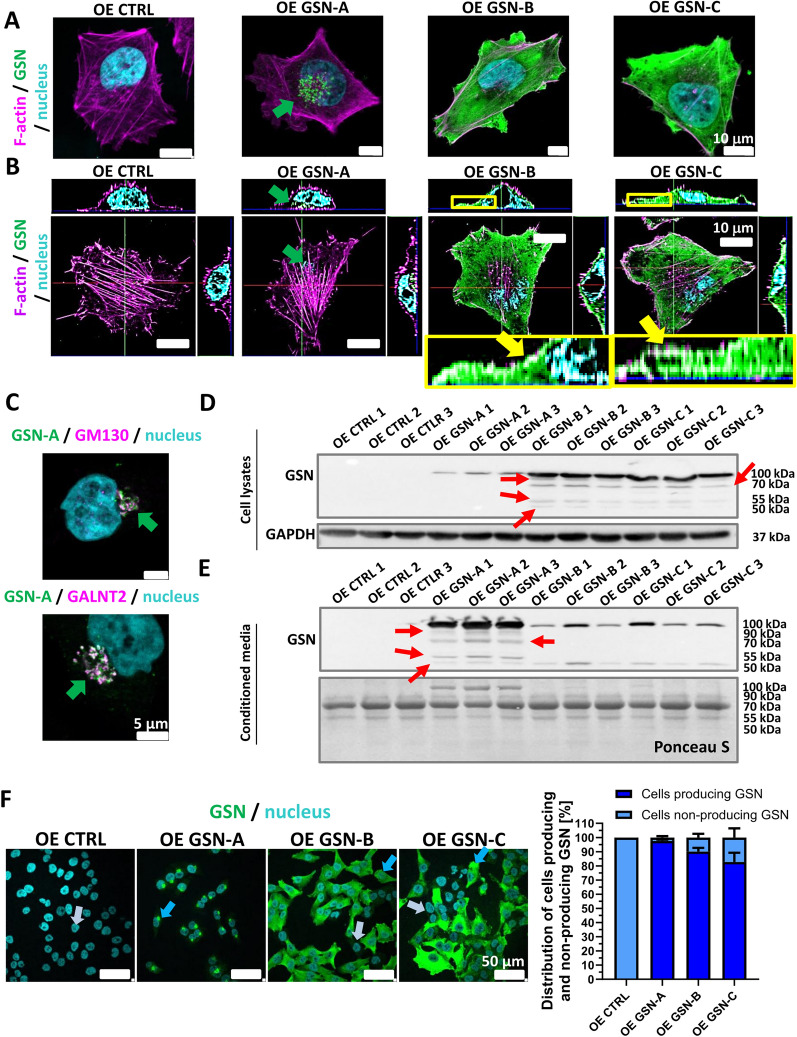
Generation of A375 cell clones producing only one GSN isoform based on A375 human skin melanoma cells with *GSN* knockout. The Materials and Methods section describes the procedure for obtaining the clones. Cells cultured for 24 h on the non-coated surface were fixed and stained. The photos were taken using a confocal microscope. **A**, **B** Images of cells stained with antibodies directed against GSN and fluorescently labeled phalloidin and Hoechst 33342 to detect GSN, F-actin and the cell nucleus, respectively. The green arrow points to GSN-A accumulation near the cell nucleus. **B** Maximum intensity projections and orthogonal sections of z-stack confocal images. The yellow arrows indicate the localization of GSN in the submembrane region. Boxed areas are shown at higher magnification. **C** Verification of the presence of the GSN-A isoform in the Golgi apparatus of the tested clones. Images of cells stained with antibodies directed against GSN and markers of the Golgi apparatus GM130 and GALNT2. The green arrows point to GSN-A aggregates near the cell nucleus. Western blot analysis of cell lysates (**D**) and conditioned media (**E**) collected from A375 cell cultures and clones producing only one GSN isoform. Protein detection was performed using antibodies directed against GSN and GAPDH. GAPDH detection and Ponceau S staining served as loading controls. 20 µg of protein was loaded into each well. The red arrows indicate the presence of GSN forms with different molecular weights. **F** Distribution of GSN-producing and non-GSN-producing cells in the OE CTRL, OE GSN-A, OE GSN-B, and OE GSN-C cell clone populations. Cells growing for 24 h on the non-coated surface were fixed and stained to detect GSN and cell nuclei. Light blue and dark blue arrows indicate non-GSN-producing and GSN-producing cells, respectively. The percentage for every group is presented in a bar section. For each group of clones, 12 photos were analyzed (at least 374 cells per condition)

The A375 cell line was selected for further studies because it produces all three GSN isoforms examined here and secretes the highest amount of GSN. An A375 cell clone that lacks GSN expression (KO *GSN*) [23] was stably transfected with a control DNA construct or genetic constructs encoding GSN-A, GSN-B, or GSN-C, respectively. As a result, three sets of three cell clones of A375 cells producing only the selected isoform of the tested protein (OE GSN-A, OE GSN-B, and OE GSN-C) and three control clones (OE CTRL) were generated. In each experiment, three cell clones from each clone group were tested. Immunocytochemical staining and Western blot analysis demonstrated correct GSN expression in the derived cell clones (Fig. 2).

Antibodies directed against total GSN and fluorescently labeled phalloidin binding to F-actin were used to assess the localization of GSN in clones producing selected GSN isoforms. Confocal images showed that GSN-A accumulated in clusters near the cell nucleus (Fig. 2A). In contrast, GSN-B and GSN-C were localized throughout the cell body. To better examine the localization of GSN isoforms in the cell, maximum intensity projections and orthogonal sections of z-stack confocal images were prepared. It was found that both GSN-B and GSN-C are localized in the cell body and submembranous area (Fig. 2B). Additionally, immunocytochemical staining of cells producing the GSN-A isoform was performed, revealing the localization of GSN and proteins that are markers of the Golgi apparatus: GM130 and GALNT2 (Fig. 2C). Signals for GSN-A, GM130, and GALNT2 overlapped, indicating that GSN-A was present in the Golgi apparatus. This was expected since GSN-A is a secretory protein.

Western blot analysis of cell lysates indicated that all tested clones, except for the control clones, expressed GSN at the protein level (Fig. 2D). For cells expressing GSN-B and -C, in addition to the bands at 100 kDa, bands at 70, 55, and 50 kDa were also observed. Surprisingly, all GSN isoforms were detected in the concentrated conditioned medium (Fig. 2E), suggesting that isoforms A, B, and C are present in the extracellular space. For all GSN isoforms tested, in addition to the band at 100 kDa, a band at 50 kDa was also present. However, only in the case of GSN-A were additional bands seen at 90, 70, and 55 kDa.

To determine whether all cells of the obtained cell clones produced GSN, immunocytochemical staining was conducted using antibodies specific to GSN. For OE GSN-A, OE GSN-B, and OE GSN-C cells, 2, 10, and 17% of the cells, respectively, exhibited no signal or a very weak signal associated with GSN (Fig. 2F).

In summary, all studied GSN isoforms localize within a cell as expected, with GSN-A in the Golgi apparatus and GSN-B and -C in F-actin-rich regions. GSN-A is secreted, while GSN-B and -C are also present in conditioned medium.

### Morphology of the A375 cells producing different versions of GSN

**Fig. 3:**
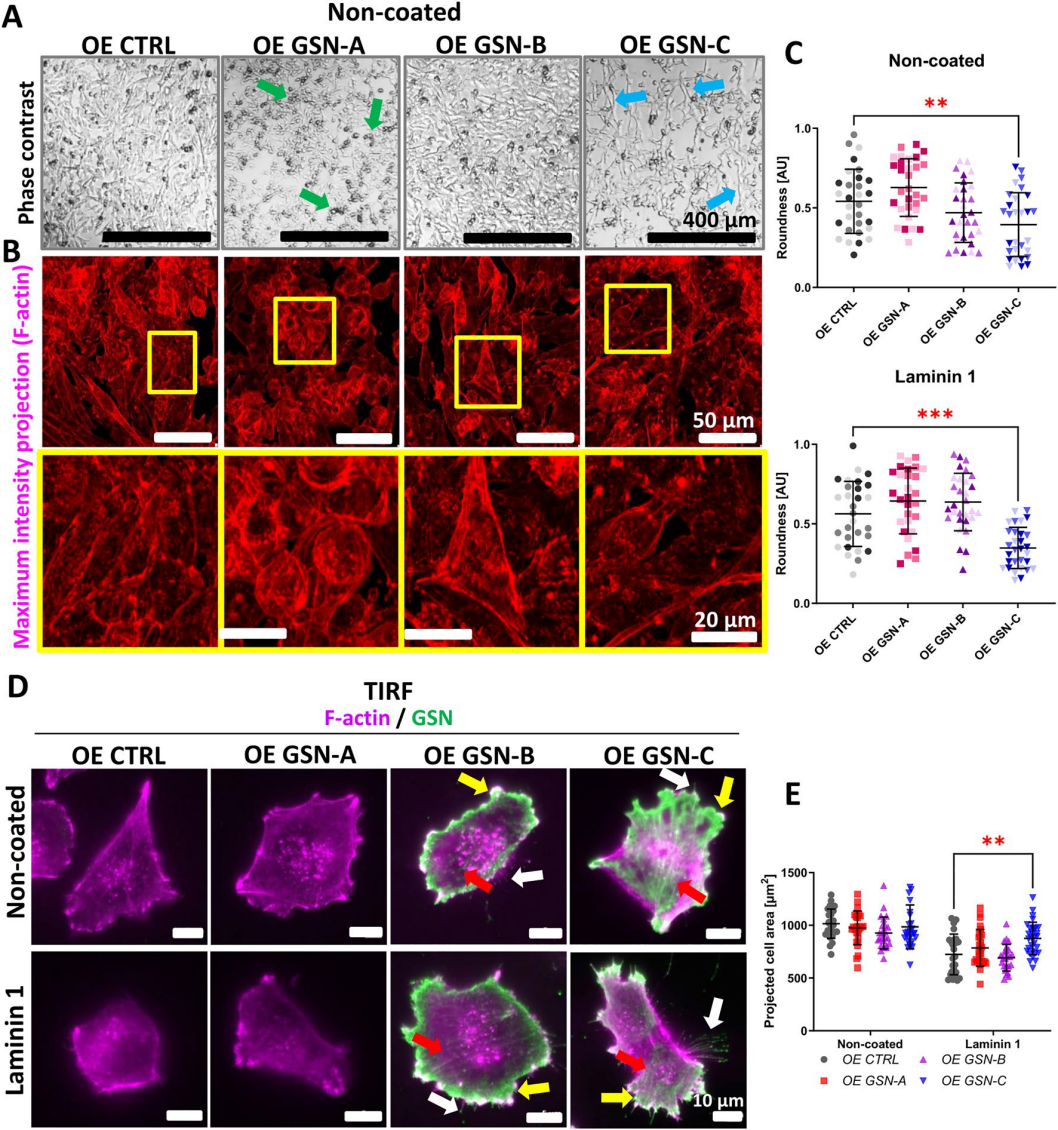
Morphological differences of A375 cell clones producing a individual GSN isoform. **A** Once the cells on the non-coated plastic surface reached full confluency, they were photographed. Green and blue arrows indicate rounded and elongated cells, respectively. **B** The analyzed A375 cell clones were grown until full confluency. Afterward, the cells were fixed and stained with phalloidin-Alexa Fluor^®^ 568 to detect F-actin. Maximum intensity projections of the captured Z-stacks are presented, with boxed areas shown at higher magnification. **C** The roundness of the cell clones growing on uncoated and laminin 1-coated coverslips was measured (n = 29–30). **D** The cells growing in an 8-well Lab-Tek dish were fixed and stained to detect F-actin and GSN. Subsequently, images were captured using TIRF microscopy to show the localization of GSN and F-actin in the submembrane region. Yellow arrows highlight the presence of GSN at the cell perimeter, while red and white arrows indicate GSN accumulation at stress fibers and in filopodia. **E** Based on the TIRF microscopy images, the projected cell area was measured (n = 25–30). Results are reported as mean ± SD; *p* ≤ 0.01 (**) and *p* ≤ 0.001 (***); assessed using two-way ANOVA and Dunnett's multiple comparison test

While culturing the obtained cell clones, we observed that the cells, upon reaching full confluency, displayed diverse morphologies among the studied cell clones. The control cells and GSN-B-producing cells exhibited spindle-like shapes, whereas the GSN-A and GSN-C-producing cells presented more rounded and elongated forms than the control cells (Fig. 3A, B). The cells that did not reach full confluency exhibited similar morphologies to those under full confluency (Fig. 3C). The GSN-C-producing cells were less round compared to the control cells. In the case of GSN-A clones, a tendency toward increased roundness was observed compared to control clones. Since melanocytes and melanoma cells grow in contact with laminin 1, which is present in the basement membrane and the melanoma environment, we included this condition in the subsequent experiments. This was particularly relevant because we previously demonstrated that A375 KO *GSN*-cells showed significantly affected motility when the KO *GSN*-clones were grown on laminin 1 or Matrigel^®^ but not on collagens or fibronectin [23]. Using Total Internal Reflection Fluorescence (TIRF) microscopy, we assessed the projected area of the cells. We noted that only the GSN-C-producing cells occupied a larger surface area than the control cells, but this occurred only when the cells were seeded on laminin 1 (Fig. 3D, E). Notably, GSN-B and -C were localized at the cell's perimeter, where they were enriched in lamellipodia colocalizing with F-actin at the leading edge. While GSN-C was also identified in filopodia, GSN-B and -C accumulated along stress fibers.

Overall, it demonstrates that GSN-B and -C are present at the plasma membrane where F-actin accumulates. However, GSN isoforms have different effects on cell morphology, with GSN-A-producing cells being more rounded, while GSN-C-producing cells are more elongated compared to control and GSN-B-producing cells.

### The influence of the individual GSN isoforms on the proliferation and potential to form tumor by A375 cells

**Fig. 4:**
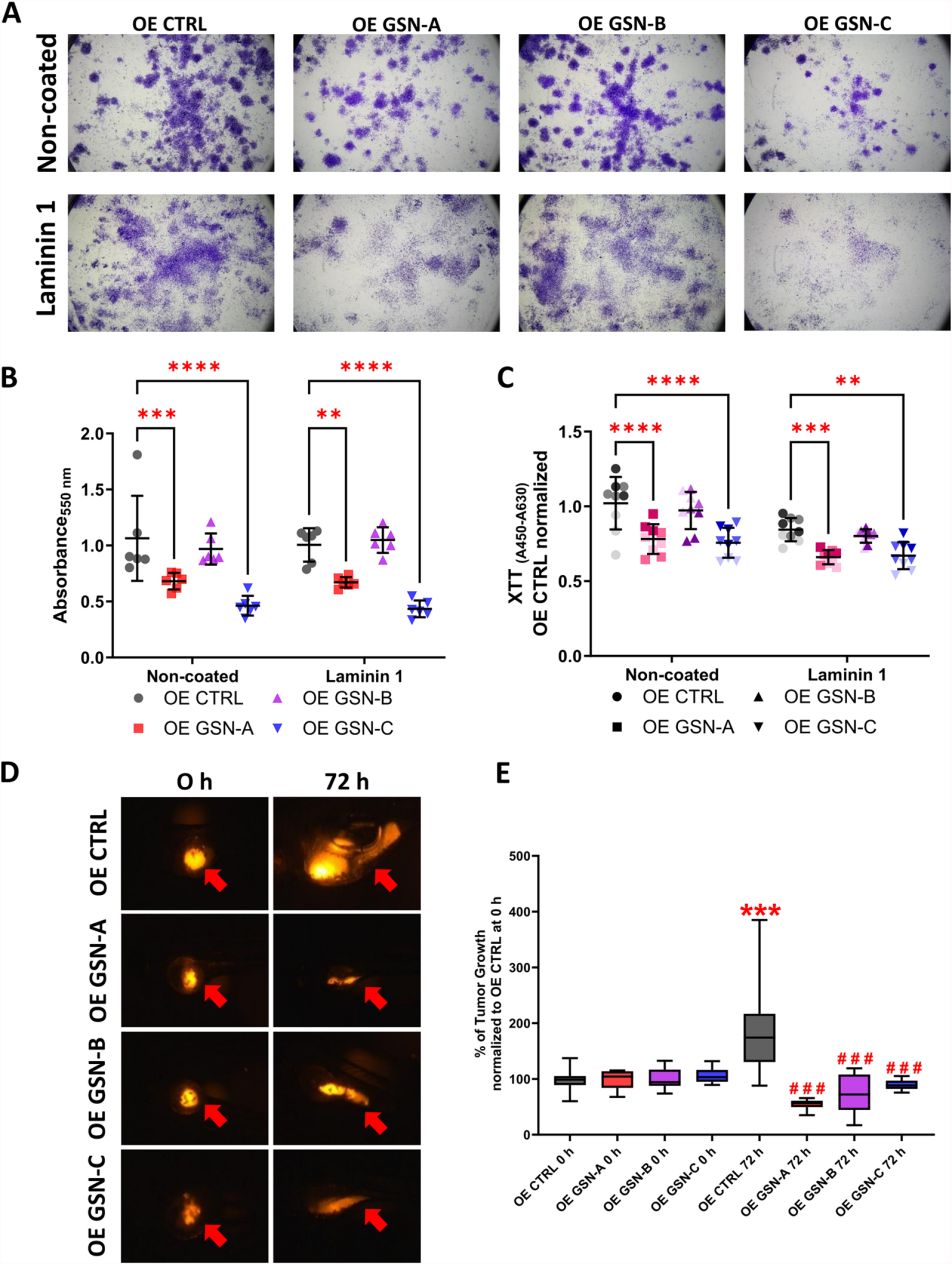
The influence of individual GSN isoform production on the proliferation rate of A375 cells. **A** A colony formation assay was conducted to assess the ability of the cells to form colonies from single cells. One week after seeding, the cells were subjected to the assay, and images of the stained colonies/cells were captured. Next, the stain was dissolved, and absorbance was measured for each condition (n = 6) (**B**). **C** An XTT assay was performed on cells cultured for 72 h on either a non-coated or laminin 1-coated surface (n = 9). **D** Stained with DiI, A375 cell clones were xenotransplanted into the yolk sac of *Danio rerio* larvae 2 days after fertilization. After capturing images at 0 h, embryos were cultured for 72 h. Then, additional images were taken. Red arrows point to fluorescently labeled cells in the yolk sacs of treated zebrafish embryos. **E** Tumor volume quantification was conducted on images taken at 0 and 72 h after introducing DiI-stained cells into the yolk sacs of zebrafish embryos (at least 5–6 embryos were analyzed per condition). Results are expressed as mean ± SD; *p* ≤ 0.01 (**), *p* ≤ 0.001 (*** or ###), and *p* ≤ 0.0001 (****); using two-way ANOVA and Dunnett's multiple comparison test. In (**E**), asterisks indicate a comparison of results obtained for OE CTRL at 72 h to OE CTRL at 0 h. Hash symbols represent the comparison of outcomes for clones producing different GSN isoforms (72 h) to OE CTRL cells (72 h)

Next, we assessed the ability of the cells to form colonies from single cells. We noted that OE GSN-A and -C clones were less effective at forming colonies than the control clones and OE GSN-B cells (Fig. 4A, B). Cells producing GSN-B formed colonies to the same extent as control cells. These observations were consistent across different coating conditions. It's important to note that the cells growing in colonies on laminin 1 were more dispersed than those growing on the non-coated surface (Fig. 4A). We assessed the mitochondrial activity of the tested cells using the XTT assay. Based on the obtained results, we concluded that OE GSN-A and OE GSN-C cells exhibited lower mitochondrial activity, which translated to a reduced proliferation rate compared to control cells when cultured on either the non-coated or laminin 1-coated surface (Fig. 4C). Using the IncuCyte system, we also evaluated the confluence of cells resulting from proliferation and the surface area occupied by the cells (Fig. S5). Analysis showed that clones producing individual GSN isoforms were less confluent when grown on a non-coated surface for 72 h. Cells cultured on the laminin 1-coated surface behaved similarly, with OE GSN-A, OE GSN-B, and OE GSN-C clones exhibiting lower confluence than control cells, and OE GSN-C displayed the lowest confluence among the tested cell clones (Fig. S5).

Finally, we xenotransplanted the studied clones into the yolk sacs of zebrafish embryos to examine their potential to develop tumors in a living organism. We observed that 72 h after introducing the cells into the embryos, only the control cells showed an increase in tumor volume over time compared to the other types of clones (Fig. 4D, E).

In summary, the data presented in this section shows that the proliferation rate of GSN-A and GSN-C-producing cells is slower compared to control cells and cells producing GSN-B. However, all types of cells producing individual GSN isoforms were incapable of forming tumors in *Danio rerio* larvae.

### Evaluation of the influence of individual GSN isoforms on the organization of actin cytoskeleton

**Fig. 5:**
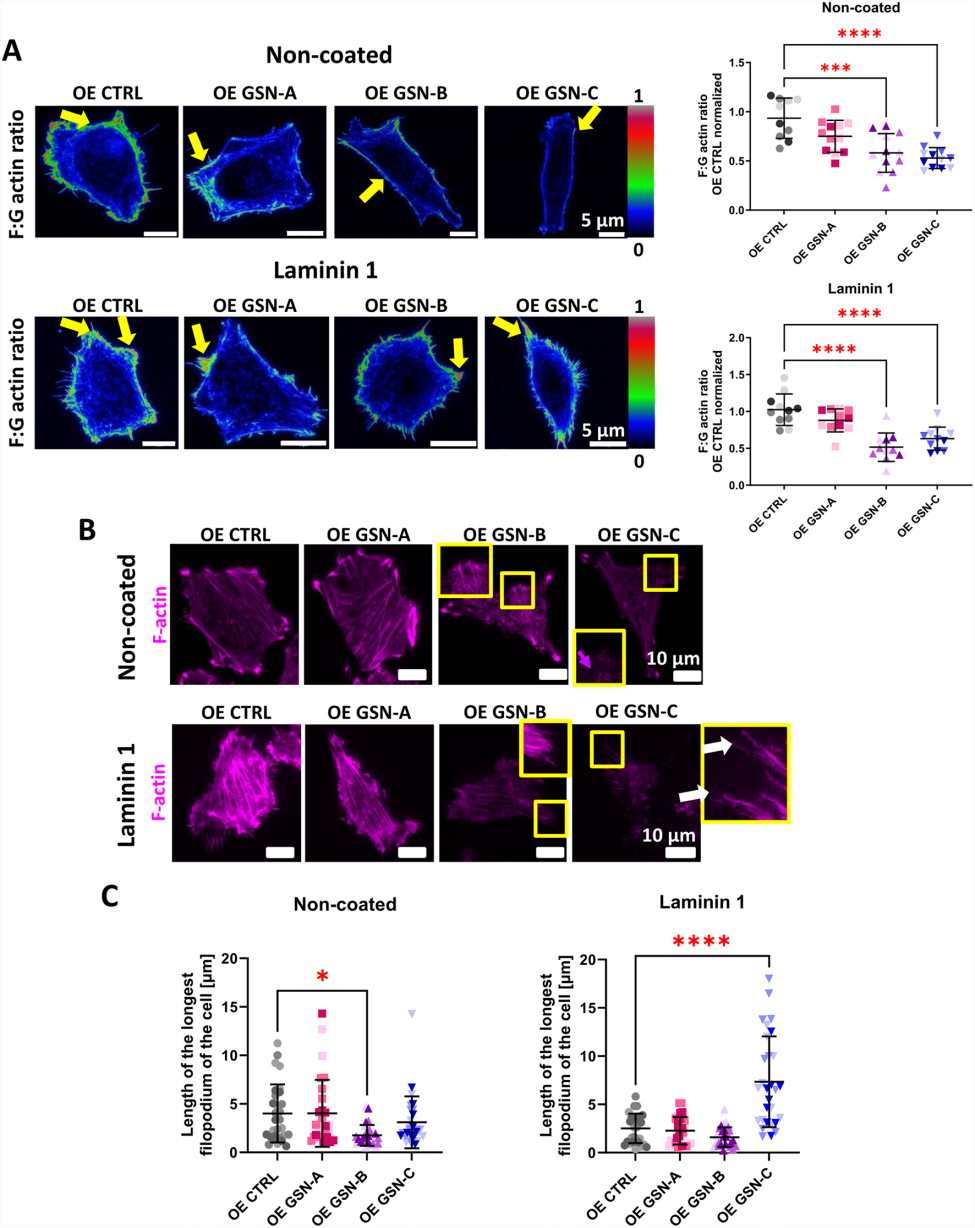
Diverging impact of GSN isoforms on actin polymerization states in melanoma cells and filopodia formation. **A** Cells incubated for 48 h on non-coated or laminin 1-coated coverslips were fixed and stained to detect G-actin and F-actin. Z-stack images were captured using a confocal microscope. Using Fiji software, a visualization of the ratio of the sum of intensities of F-actin to G-actin in the cells was prepared. Color coding: black indicates minimal values, while red indicates maximal values. A quantitative analysis of the average F-actin to G-actin intensity ratio per cell was performed using Fiji software (n = 12). Yellow arrows mark the cell perimeter where the F:G actin ratio was highest. Images of single channels showing either F- or G-actin are displayed in Fig. S6. **B** Cells producing individual GSN isoforms, incubated for 48 h on non-coated or laminin 1-coated coverslips, were fixed and stained to detect F-actin. Images were captured through confocal microscopy with consistent settings to show the reduced signal for F-actin in cells producing GSN-B or -C. Boxed areas are displayed at higher magnification. **C** Based on the microscopic images presented in (**B**), the average length of the longest filopodium in the cells was calculated (n = 30). Results are presented as mean ± SD; *p* ≤ 0.05 (*), *p* ≤ 0.001 (***), and *p* ≤ 0.0001 (****); two-way ANOVA and Dunnett's multiple comparison test

Initially, we decided to examine the state of actin polymerization by calculating the ratio of F-actin to G-actin in the tested cells. We observed that OE GSN-B and OE GSN-C cells exhibited a lower F:G actin ratio compared to the control when grown on an uncoated substrate (Fig. 5A, S6). Similar results were found for cells growing on a surface coated with laminin 1. Cells producing GSN-B or GSN-C displayed a lower F:G actin ratio than those producing GSN-A and the control cells. Interestingly, for OE GSN-A, the F:G actin ratio was lower than in the control cells, although it was not statistically significant. In all clones, F-actin was predominantly located at the cell periphery/in the plasma membrane region (Fig. 5A and S6), while G-actin was primarily found in the perinuclear area (Fig. S6).

Next, we decided to examine the organization of F-actin at the plasma membrane in the studied clones. The data presented clearly show that OE GSN-B and OE GSN-C cells are characterized by significantly lower levels of F-actin at the plasma membrane (Fig. 5B). Furthermore, we observed that the cells producing GSN-C had very long filopodia when cultured on laminin 1. An analysis of the length of the longest filopodium per cell revealed that OE GSN-B cells growing on a non-coated surface exhibited shorter filopodia compared to control cells and those producing GSN isoform A (Fig. 5C). In contrast, OE GSN-C cells growing on a laminin 1-coated surface formed the longest filopodia among all tested clones, with sizes varying between 2 and 20 µm.

In conclusion, it can be stated that both GSN-B and GSN-C play a fragmenting role on F-actin, resulting in a decreased F:G actin ratio. Furthermore, GSN-C-producing cells growing on laminin-1 exhibit relatively long filopodia.

### The role of individual GSN isoforms in melanoma cell motility

**Fig. 6:**
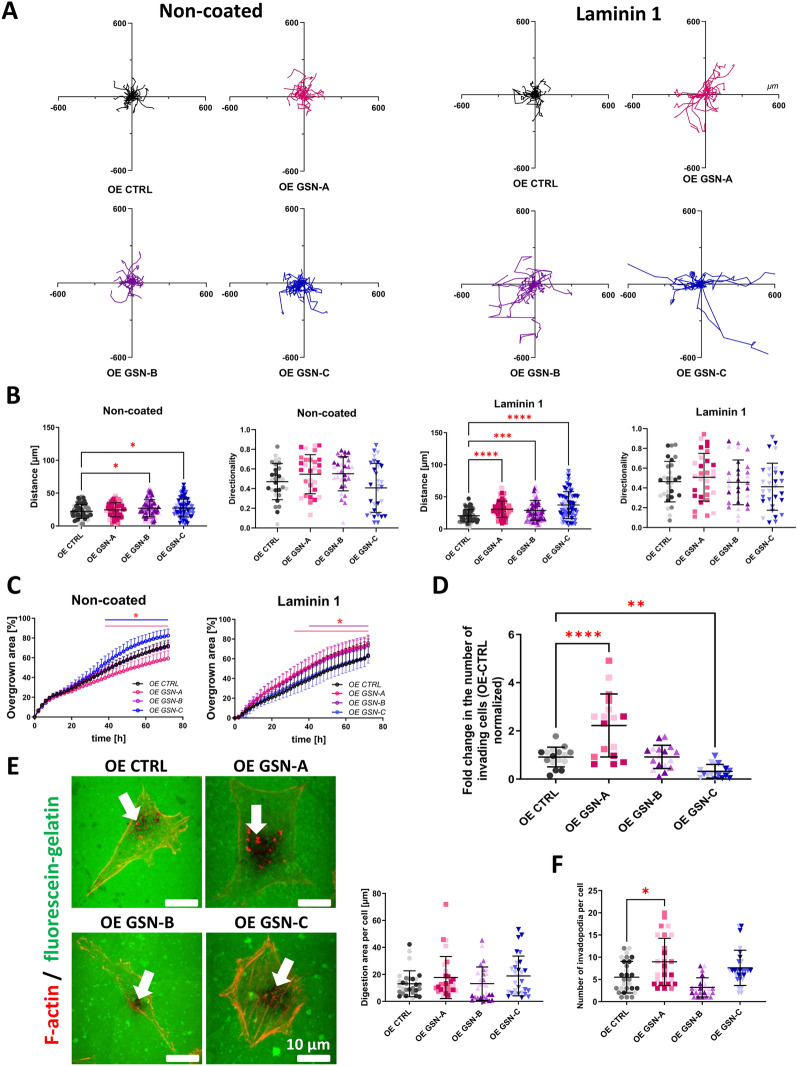
Isoforms of GSN have different impacts on the movement of melanoma cells. Cells were cultured on either a non-coated or laminin 1-coated surface in the ImageLock 96-well plate. Over 24 h, the cells were monitored using the IncuCyte system. Subsequently, (**A**) the movement trajectories of single cells (n = 30) were plotted, and (**B**) the distance (n = 90) and directionality of migrating cells (n = 30) were calculated. **C** An analysis of collective migration of the cells on non-coated versus laminin 1-coated surfaces was conducted. After creating a scratch with the WoundMaker tool, cells were monitored with the IncuCyte system for 72 h, taking photos every 2 h. The scratch surface covered by cells was counted over time and presented in graphs as a percentage of the overgrown area (n = 9). **D** The studied A375 cell clones underwent the invasion/3D migration assay (n = 18). **E** Assessment of the ability of A375 clones to digest the extracellular matrix. The cells seeded onto gelatin-fluorescein-coated coverslips were fixed and stained with fluorescently labeled phalloidin to detect F-actin. White arrows indicate invadopodia with digestion potential. Dark spots represent digested fluorescent gelatin. The fluorescein-gelatin digestion area and (**F**) the number of invadopodia co-localizing with the degradation area per cell were evaluated (n = 30). Results are presented as mean ± SD; *p* ≤ 0.05 (*), *p* ≤ 0.01 (**), *p* ≤ 0.001 (***), and *p* ≤ 0.0001 (****); two-way ANOVA and Dunnett's multiple comparison test

Because changes in the F:G actin ratio in a cell are directly associated with alterations in motility [26], and since GSN has been shown to affect the two-dimensional movement of A375 cells [23], migration assays were conducted using clones that produce individual GSN isoforms to investigate which GSN isoform is involved in this process. Spontaneous migration of single cells was monitored based on the trajectory of cell movement over 72 h (Fig. S7A). However, it was necessary to shorten the tracking time of cells to 24 h (Fig. 6A) due to the migration of GSN-C-producing cells growing on laminin out of the field of view. The distance covered by the cells during observation and the directionality of their movement were calculated. It was observed that GSN-B and -C-producing cells covered the longest distance, averaging 25.4, and 26.66 µm, respectively, when migrating on the non-coated surface, compared to other cells, which covered averages of 20.66, and 23.2 µm for OE CTRL, and OE GSN-A cells, respectively (Fig. 6B). For cells cultured on laminin 1, GSN-A, -B, and -C-producing cells covered greater distances than the control cells. GSN-C-producing cells exhibited the highest migratory potential (37.4 µm) compared to the other cells and covered almost twice the distance compared to the control cells. In contrast, cells producing GSN-A or -B migrated about 30% better than the control cells (30.6 and 29 vs. 20.5 µm). No differences were observed in the directionality of spontaneous movement among the analyzed cells under the tested conditions (Fig. 6B). When cells were observed for 72 h, those producing the b isoform of GSN traveled a greater distance than the control. However, when cultured on a laminin 1-coated surface, OE GSN-Clones A, B, and C covered longer distances than the control cells (Fig. S7A). The collective migration ability of the studied cells was analyzed in the following step. The collective migration test indicated that OE GSN-C cells growing on the non-coated surface covered the scratch faster, while OE GSN-A clones covered it more slowly than the control cells (Fig. 6C, S7B). Conversely, an opposite result was noted for movement on a surface coated with laminin 1, where cells producing GSN-A or -B migrated faster than GSN-C-producing cells and control cells (Fig. 6C, S7B).

Our previous studies have shown that GSN affects the 3D movement of melanoma cells [23], so we decided to investigate the role of GSN isoforms in this process. It was observed that cells expressing GSN-A exhibited approximately twofold higher invasive potential compared to OE CTRL (Fig. 6D). Cells producing GSN-C showed reduced invasive potential in comparison to control cells. To investigate the activity of invadopodia, i.e., structures involved in 3D migration, an assay evaluating the digestion activity of fluorescently labeled gelatin was utilized. It was demonstrated that the production of individual GSN isoforms did not influence the activity of gelatinases (Fig. 6E). However, GSN-A-producing cells were characterized by a greater number of active invadopodia compared to control cells (Fig. 6F).

In summary, GSN-A appears to be significant for the invasiveness of the cells, while GSN-B and GSN-C are essential for the cells’ motility in 2D.

### GSN isoforms differently regulate the adhesion abilities of cells

**Fig. 7:**
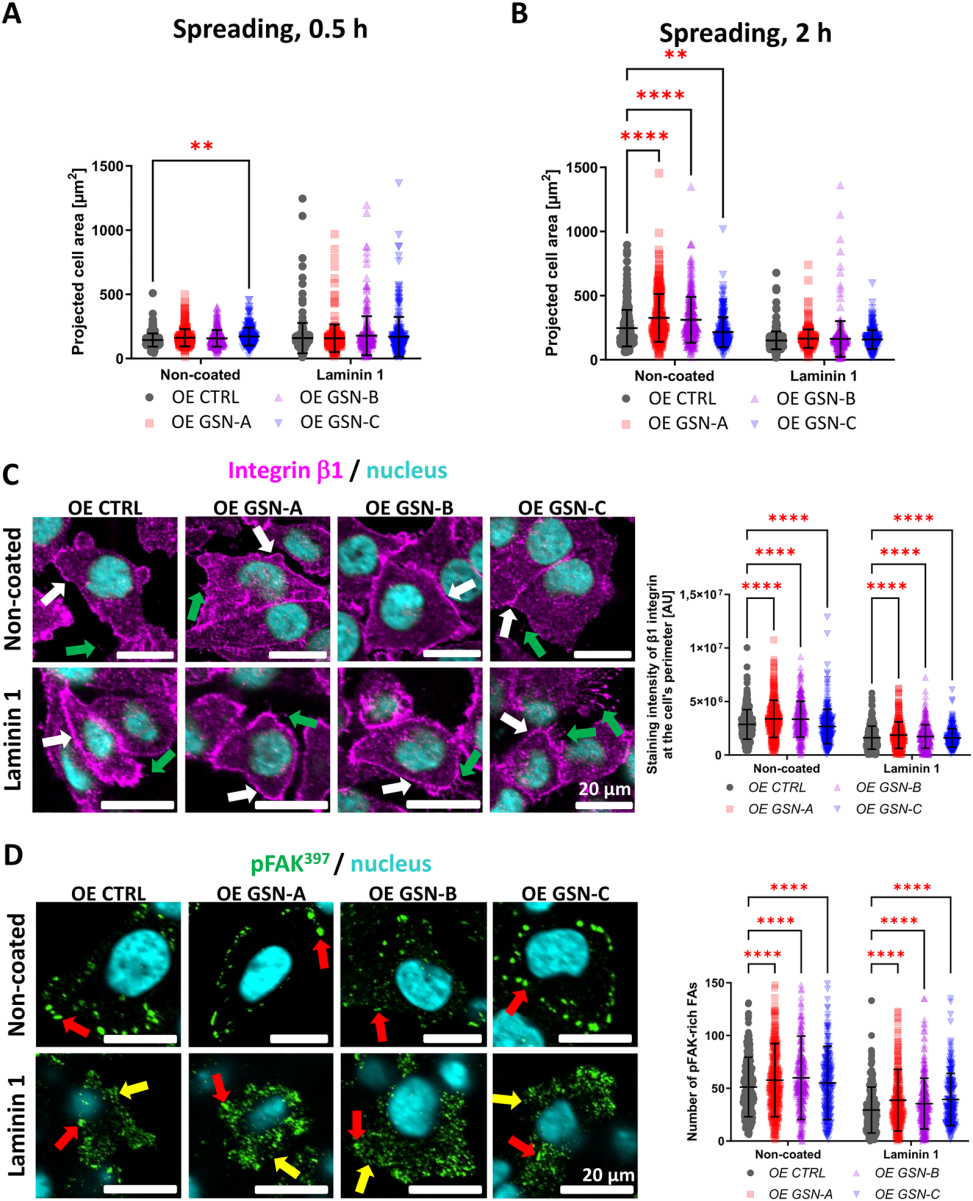
A375 cell clones producing different GSN isoforms exhibit varying spreading dynamics, distribution, and activation levels of proteins involved in adhesion. The cells were seeded into non-coated and laminin 1-coated wells of a PhenoPlate 96-well plate (Revvity). After 30 min (**A**) or 2 h (**B**), the cells were washed twice with PBS, fixed, and stained to detect F-actin. Based on the images acquired with the Opera Phenix Plus HCS system (Revvity), shown in Fig. S9, the projected area of the cells was measured using Harmony software (Revvity) (n = 300). **C–D** Cells incubated for 48 h on either the non-coated or laminin 1-coated surface in a PhenoPlate 96-well plate (Revvity) were fixed and stained to detect either β1 integrin (**C**) or FAK phosphorylated on Tyr397 (active FAK) (**D**). Images were acquired using the HCS system. Quantitative analysis of fluorescence intensity corresponding to β1 integrin staining in the submembrane area of A375 cell clones (Fig. S10A-A’) was performed with Harmony software (Revvity) (n = 300). White arrows indicate the localization of β1 integrin within the plasma membrane, while green arrows show the presence of β1 integrin along filopodia. In the case of staining with anti-pFAK^397^ antibodies, the number of spots meeting the set threshold (Fig. S10B-B’) was quantified (n = 300). Red and yellow arrows highlight prominent pFAK^397^ aggregation in focal adhesions and small puncta, respectively. Results are presented as mean ± SD; *p* ≤ 0.01 (**) and *p* ≤ 0.0001 (****); two-way ANOVA and Dunnett’s multiple comparison test

Because we observed that the contact area of OE GSN-C cells with the substrate was larger than that of control cells when grown on laminin 1 (Fig. 3E), we chose to evaluate the cells' adhesion potential and spreading dynamics. We conducted the adhesion assay and found that only OE GSN-C cells adhered to uncoated or laminin 1-coated surfaces in reduced amounts compared to control cells (Fig. S8). Next, to examine the spreading abilities of the cells in more detail, we seeded the cells into the wells of a 96-well plate and, after 30 min or 2 h, fixed and stained them to detect F-actin (Fig. S9). Images were obtained using a High-Content Screening (HCS) microscope and automatically analyzed with software designed for processing data generated by the HCS system. 30 min after seeding the cells only OE GSN-C-producing cells exhibited a larger projected cell area than control cells (Fig. 7A), but only when the cells adhered to the uncoated surface. After 2 h, the surface area occupied by a cell was larger only for OE GSN-A and -B clones compared to control clones (Fig. 7B). However, there were no differences among cell clones when they grew on a laminin 1-coated surface.

Because GSN is known to regulate the inside-out activation of β1 integrin [27, 28], we evaluated the aggregation of β1 integrin at the plasma membrane in the studied clones. It was observed that this integrin accumulated at the plasma membrane under all tested conditions (Fig. 7C). Furthermore, we observed the presence of β1 integrin along filopodia, which was anticipated [29]. It is noteworthy that 1 integrin in certain melanoma cell lines, including A375 cells, does not localize in focal adhesion (FA) as, for example, αV3 integrin does (data not shown). Nonetheless, we observed that in OE GSN-A and -B cell clones, β1 integrin was more abundant at the plasma membrane than in control cells when the cells were cultivated under both coating conditions. In contrast, there was a reduced signal at the plasma membrane for OE GSN-C cells on both non-coated and laminin 1-coated surfaces. In the non-coated condition, these data reflect the results of the projected cell area 2 h after seeding the cells.

Finally, we examined the active form of focal adhesion kinase (FAK) to assess the number of pFAK^397^-rich spots representing mature FAs. We observed a higher number of pFAK^397^-rich spots (FA) in OE GSN-A, -B, or -C cell clones on both non-coated and laminin 1-coated surfaces (Fig. 7D). Interestingly, for the cells growing on the laminin 1-coated surface, we noted the presence of small pFAK^397^-rich spots that were not counted due to the set threshold for identifying FAs at the perimeter of a cell (Fig. S10B–B’). This was due to their size and localization, which did not match the characteristics of mature FAs.

Overall, the data presented in this section indicate that all studied GSN isoforms are crucial for cell spreading on a non-coated substrate, but not on laminin 1. GSN-A and GSN-B are essential for the accumulation of β1 integrin at the plasma membrane, while GSN-C is not. Conversely, in GSN-C-producing cells, we observe the accumulation of β1 integrin along long filopodia when growing on laminin 1. The induced production of all GSN isoforms positively influences the activation of FAK, suggesting that all studied isoforms regulate the formation of focal adhesions.

## Discussion

The results showed that the transcripts for GSN's A, B, and C isoforms are present in various human skin melanoma cell lines and in normal and melanoma tissues. Western blotting analysis of conditioned media demonstrated the presence of GSN in the medium of the melanoma cell lines studied. Unfortunately, specific antibodies recognizing individual isoforms of GSN are not available; therefore, to determine the nature of the effects of selected isoforms of GSN on melanoma cell biology, cell clones were obtained from GSN-deprived (KO *GSN*) A375 cells [23] that exogenously produced only the protein's A, B, or C isoform—OE GSN-A, OE GSN-B, and OE GSN-C cell clones. We selected A375 cells because this cell line produces all three GSN isoforms.

All previously published results indicate the secretion of one GSN isoform through the classical pathway, which possesses the signal sequence of GSN-A [30]. Our studies also confirm the secretion of GSN-A, which was observed in the conditioned medium from melanoma cells. Its localization within the cell was demonstrated in the same areas where proteins characteristic of the Golgi apparatus—GM130 and GALNT2—localize, thereby confirming the classical secretion pathway of this protein [31]. The GSN-B and -C isoforms were found within the cell, specifically in the cell nucleus and in the plasma membrane region. They were observed to localize in structures rich in F-actin, such as filopodia, invadopodia, and stress fibers. Additionally, GSN-B and -C isoforms were detected in the conditioned medium. This may be related to the presence of GSN in extracellular vesicles, as recent studies have shown that melanoma cell exosomes contain GSN [32]. Western blot analysis of the collected media revealed additional bands at approximately 50 kDa in conditioned medium gathered from OE GSN-A, OE GSN-B, and OE GSN-C cell cultures, as well as bands at 70 and 55 kDa in GSN-A-producing cells, which may result from cleavage of GSN by proteases such as furin [33], MMP-9 [34], MMP-1, MPP-2, MPP-3, or MPP-14 [35]. Proteolysis of GSN inactivates the protein during actin depolymerization. Consequently, when GSN cleavage occurs in the extracellular space, it may lead to pathological conditions caused by extracellular actin, such as endothelial damage, respiratory distress syndrome, liver necrosis, or septic shock [35]. Future studies should focus on the influence of cleaved forms of GSN present in the ECM on melanoma cells.

Interactions among melanoma cell receptors, such as integrin receptors [36] or the non-integrin 67-kDa laminin receptor [37], and laminin [38] are essential for tumor cell adhesion, migration, and invasion [36, 37]. This suggests that laminins play an essential role in tumorigenesis. Our previous studies have shown that the most significant effect of the absence of GSN production on A375 cell migration was observed in cells growing on laminin 1 [23]. The results we described suggest a significant role of GSN in the interaction between melanoma cells and laminin 1, as cells lacking GSN production showed a markedly reduced potential for spontaneous and collective migration compared to control cells on a laminin 1-coated surface. Consequently, we decided to test the generated clones on non-coated and laminin 1-coated surfaces.

The results we obtained point to the distinct roles played by individual GSN isoforms. For instance, based on the results from the XTT test, colony formation assay, and confluency assessment, we conclude that the sole production of GSN-A and -C negatively impacts the clonogenicity and proliferation of melanoma cells. In a different type of tumor, hepatocellular carcinoma, GSN has been shown to promote the proliferation of cells [39]. On the other hand, GSN-C-producing cells exhibited significantly higher spontaneous and collective migration potential on the non-coated surface compared to control cells, and the highest spontaneous migration potential among the tested cells on the laminin 1-coated surface. Thus, GSN-C appears to be the isoform with the most potent effect on the 2D migration of A375 cells, whereas GSN-A and GSN-B enhance cell migration potential only when cells are in contact with laminin 1. Interestingly, GSN-A was found to disrupt the collective migration of cells grown on the non-coated surface.

Although in our previous study we did not study an effect of *GSN* knockdown on the actin polymerization state in the cell, we did demonstrate that it altered the formation of F-actin-rich structures [23]. Here, we found the importance of GSN's -B and -C isoforms for the actin polymerization state. This is reflected, for example, in the fact that GSN is involved in filopodia formation in A375 cells [23]. Previous studies have demonstrated that filopodia are crucial for cancer cells, as their formation is linked to metastasis development [40]. Interestingly, when cells were cultured on a laminin-1-coated surface, the absence of *GSN* expression led to a significantly greater number of filopodia, along with their increased length and density compared to control cells [23]. The improved activity of filopodia was linked to decreased motility of KO *GSN* cells on laminin 1. Research on the impact of producing selected GSN isoforms on the length of formed filopodia revealed that cells expressing only the GSN-B isoform, when cultured on a surface coated with laminin 1, exhibited reduced filopodia length compared to those expressing GSN-A or GSN-C, which is contrary to the effect seen in cells lacking *GSN* expression. Conversely, GSN-C was found to positively influence the length of filopodia in cells grown on a laminin 1-coated surface. Notably, hippocampal cells from mice with diminished GSN expression were observed to form significantly more filopodia along neurites compared to controls [41]. Analysis of individual filopodia revealed no differences in their elongation rates; however, the loss of GSN expression adversely affected their retraction phase [41]. Therefore, the reduced ability of GSN-deprived melanoma cells to migrate in 2D on a laminin-1-coated surface may be linked to the impaired retraction of filopodia caused by the lack of GSN. In contrast, GSN-C-producing cells, which exhibit greater filopodia activity, demonstrated a significantly enhanced migration potential under 2D conditions compared to the other cells. GSN-C may be an isoform of GSN that plays a role in the formation and proper functioning of filopodia, thereby improving cell mobility under 2D conditions. This hypothesis is worth testing in further studies.

We have previously demonstrated that GSN resides in the invadopodia of melanoma cells [21], where it interacts with the Arp3 protein, which, as a component of the Arp2/3 complex, binds actin [42]. GSN-deprived cells formed more invadopodia when grown on a gelatin-fluorescein-coated surface compared to control cells; however, these invadopodia were less active than those in control cells [23]. GSN is crucial for the proper functioning of invadopodia, which are F-actin-rich structures involved in cell movement under three-dimensional conditions, as we observed a decrease in the invasive potential of cells lacking GSN production [23]. The number of cells capable of migrating under 3D conditions in a gel that resembles the skin basement membrane in its composition decreased by over 60%. We have previously observed a similar situation when studying A375 cells that do not express, -actin, or actbl2 [43, 44]. These cells also displayed a reduced potential for migration under 3D conditions but formed more invadopodia. The increased number of invadopodia formed by GSN-A-producing cells on the gelatin-coated substrate correlates positively with the invasive potential of melanoma cells. OE GSN-A clones exhibited a significantly higher migration potential in the polymerized Matrigel™ layer compared to the other cells. Conversely, the observed increased invasive potential of OE GSN-A cells is not a result of enhanced digestion of the extracellular matrix, as this was not demonstrated here. However, it should be noted that the gelatin degradation assay only measures gelatinase activity, and melanoma cells can also produce collagenases [45, 46], the activity of which was not analyzed. An increased number of invadopodia is often not linked to enhanced potential for 3D migration or ECM digestion because the invadopodia that are formed may lack proteolytic activity [47]. We assume that the phenomenon observed in this work is a compensatory mechanism initiated by cells with reduced 3D migration capabilities. Recently, another actin-binding protein, cofilin, has been shown to be involved in the maturation and stabilization of invadopodia [42]. After knocking down the expression of the gene encoding cofilin, the resulting invadopodia exhibited reduced proteolytic activity and a significantly shorter duration compared to the control group. Our studies indicate that GSN, which localizes to invadopodia, influences the formation and function of these structures. However, additional studies are needed to clarify its role in these processes. Based on the results presented, it can be concluded that cells producing the A isoform of GSN exhibited a significantly higher 3D migration potential compared to the other cells.

The results indicating the involvement of GSN-A in the invasion of A375 cells align with previous observations that show an increase in the level of secretory GSN in colon cancer cells forming distant metastases in patients with colon cancer [18], and with other types of tumors [48–50]. However, because the antibodies used in those studies recognize all isoforms of GSN, and our results indicate the presence of isoforms B and C of GSN in the conditioned medium, we cannot be sure that the subject of the above studies was exclusively GSN-A. The mechanism by which isoform A may affect cell invasion and migration is unknown. However, it is hypothesized that in the extracellular environment, GSN-A may interact with other proteins, thereby influencing cell behavior. Such interactions could be important for intracellular signaling and cell movement, which would align with previous reports indicating the interaction of GSN with fibronectin [52] or with β2 glycoprotein I and α5β1 integrin [53]. On the other hand, it was reported that GSN-A can interact with secreted sphingosine-1-phosphate (S1P), scavenging it in the ECM [54], and thus blunting the activation of S1P receptors—S1PRs, which regulate cell migration, proliferation, and F-actin organization [55].

To date, there are very few reports on the role of GSN in adhesion. However, it has been shown that this protein is crucial for the intracellular activation of β1 integrin and, therefore, for the adhesion of murine acute lymphocytic leukemia cells [27]. Our previous studies indicate direct interactions between GSN-B and RPSA, a ribosomal protein that likely acts as a non-integrin receptor for laminin [22]. In addition, there are reports of plasma GSN interacting with integrin α5β1 [53]. However, the mechanism of action of GSN in adhesion is not understood. Studies show that GSN can directly interact with some FA proteins, such as FAK or Src kinase, as reviewed elsewhere [56]. Nevertheless, the exact mechanism of these interactions is unknown. The formation of mature FA is associated with the activation of FAK kinase [57]. Although increased number of pFAK^397^-rich FAs were observed in GSN-A, -B, and -C expressing cells, only OE GSN-C cells exhibited changes in adhesion potential. We found that cells producing GSN-C were characterized by reduced adhesion capacity and the lowest β1 integrin signal at the plasma membrane across all tested clones on uncoated and laminin 1-coated surfaces. Furthermore, OE GSN-C cells had a more elongated shape and a smaller projected cell area when grown on a laminin-coated surface compared to control cells, which also suggests altered adhesive properties of these cells.

We recognize that in a different melanoma cell line with a varying genetic background, our experimental setup could yield divergent outcomes. However, we are convinced that the non-redundant functionality of GSN isoforms could still be observed. One of the questions that remains to be answered is what mechanisms are underlying the different effects of individual GSN isoforms within the analyzed parameters. In the case of secreted GSN-A, the effect on the invasion and proliferation abilities of melanoma cells could be mediated by S1P-S1PRs or the interaction with the external part of the adhesion complex. Yet, the exact mechanism driving the impact of GSN-A on melanoma cells remains unclear. The effects of different GSN-B and GSN-C isoforms on migration, proliferation, and morphology of the cells involve a more complex understanding of their causes. There are no studies focusing on a direct molecular comparison of these isoforms. The additional amino acids at the N-terminus of GSN-C likely influence its secondary and tertiary structures, which could translate into, for example, varying affinities for microfilaments or different interaction partners compared to GSN-B. Finally, it would be interesting to investigate whether dual combinations, such as GSN-B and GSN-C or GSN-A and GSN-B, yield different outcomes.

## Conclusions

Since our previous study has shown that high levels of GSN production are associated with the progression of cutaneous melanoma [22], research aimed at understanding the role of individual isoforms of this protein in melanoma biology is of great importance. Experiments conducted on cells lacking *GSN* expression have demonstrated that the isoforms of this protein do not perform the same functions in skin melanoma cells. Melanoma cells were found to produce and secrete all the GSN isoforms studied, although only the A isoform of GSN possesses a signal sequence indicating protein secretion. It was observed that both GSN-B and GSN-C affect the actin polymerization state and localize in structures rich in F-actin. All the GSN isoforms studied participate in both individual and collective migration of melanoma cells. However, cells producing GSN-C exhibited the greatest migration potential under 2D conditions. Our study shows that GSN isoforms are not redundant in their effects on the actin cytoskeleton and their role in cell motility. We found that in skin melanoma tissue, the highest amount of transcript among the studied GSN isoforms was noted for GSN-B, which could imply that this isoform is more important for melanoma progression than GSN-A and GSN-C. Nonetheless, our outcomes, including those from experiments on zebrafish embryos, indicate that a mixture of GSN isoforms seems to be responsible for the proper functioning of melanoma cells, enabling them to maintain both an appropriate proliferation rate and invasive potential.

## Methods

### Cell culture conditions and cell treatments

A375 (Cellosaurus #CVCL_0132) and Hs294T (Cellosaurus #CVCL_0331) cell lines were obtained from ATCC^©^, while WM1341D (Cellosaurus #CVCL_6787) and WM9 (Cellosaurus #CVCL_6806) cells came from Rockland Immunochemicals Inc. Dulbecco’s modified Eagle’s medium (with a reduced concentration of NaHCO_3_ at 1.5 g/l) was supplemented with 10% FBS, 1% L-Glutamine, and 1% Antibiotic–Antimycotic from Thermo Fisher Scientific. The cell lines were free of Mycoplasma and cultured following the suppliers’ recommendations. Cells were maintained at 37 °C in a humidified atmosphere of 5% CO_2_ and split twice a week. A375 cells with *GSN* knock-out and control clones were generated using the CRISPR/Cas9(D10A) technique and are described elsewhere [23, 58]. Normal human melanocytes from adult donors (NHEM-Ad) were obtained from Lonza and cultured in MGM™−4 Melanocyte Growth Medium-4 BulletKit™ according to the manufacturer. Only early passage NHEMs were used for the analysis to prevent dedifferentiation cells.

### Patient samples and immunohistochemistry (IHC)

Healthy skin fragments were collected from patients who underwent surgery for non-skin-related diseases. Patients with cutaneous melanoma who were diagnosed and treated at the Regional Oncology Centre in Opole, Poland, between 2005 and 2010 were included in the study. Enrollment of patients was based on the availability of medical documentation and paraffin blocks containing primary skin tumors. This study was conducted in accordance with The Code of Ethics of the World Medical Association (Declaration of Helsinki) related to experiments involving humans, and patient consent was obtained. The study received review and approval from the ethics committee at Wroclaw Medical University, Poland (No 580/2019). Tissue microarrays (TMAs) comprising three 1.5 mm tissue cores from each tumor were created automatically (TMA Grand Master, Sysmex). Immunohistochemical analysis was performed using rabbit polyclonal anti-GSN-Antibody (Abcam) or rabbit monoclonal anti-Sox10 antibody (Cell Marque Antibodies) on 4-μm-thick paraffin sections mounted on silanized slides (Agilent DAKO, Santa Clara, CA, USA). The slides underwent automated dewaxing, rehydration, and heat-induced epitope retrieval with EnVision Target Retrieval Solution (Agilent DAKO) for 30 min at 97 °C in the PT Link Pre-Treatment Module for Tissue Specimens (DAKO). Liquid Permanent Red (Agilent DAKO) was used as the detection system. Negative controls were processed using FLEX Rabbit Negative Control, Ready-to-Use, instead of the primary antibody. The antibodies used and their dilutions are listed in Table S1.

### Evaluation of transcript levels in normal skin and human melanoma samples

Transcript expression data were downloaded from UCSC Xena [59]. More specifically, UCSC TOIL RNA-seq recomputed data processed with RSEM expected counts (version 2016-09-02) were used [60]. For downstream analyses, we used a subset of the UCSC TOIL recomputed data, which included GTEX skin – 555 RNA-seq profiles of skin obtained from healthy individuals; TCGA SKCM – 469 RNA-seq profiles of human skin cutaneous melanoma obtained from The Cancer Genome Atlas (TCGA) [61, 62]. GSN transcripts were annotated using the GENCODE version of the human genome (Release 23/GRCh38.p3) [63]. Specific GSN transcripts were assigned to three GSN isoforms (A, B, and C) using the archived Ensembl database (version 80) [64]. For the GSN-B isoform consisting of three transcripts, rounded RSEM expected counts were summed. Subsequently, the entire dataset was transformed to log2(Transcripts per Million + 1) values using the *CustomSelection* package [65]. All statistical analyses were performed in R (version 4.4.2). Violin plots were generated using the *ggpubr* (Kassambara A (2023). ggpubr:'ggplot2'Based Publication Ready Plots. R package version 0.6.0.999, https://github.com/kassambara/ggpubr) and *tidyverse* [66] packages. TPM levels of GSN isoforms between healthy and tumor tissues were compared using the Kruskal–Wallis test. A *p*-value ≤ 0.05 was considered statistically significant.

### Xenotransplantation of the cells into *Danio rerio* larvae

The cells were suspended at a density of 1 × 10^6/ml and then labeled with Vybrant^™^ DiI Cell-Labeling Solution (Thermo Fisher Scientific) at a concentration of 5 µl/ml, following the manufacturer’s instructions. Two days post-fertilization (dpf), zebrafish embryos were anesthetized with 0.003% tricaine (Sigma-Aldrich, St. Louis, MO, USA) and placed on a 10 cm Petri dish coated with 1% agarose. The labeled cell suspension was introduced into the yolk sac via microinjection in 2 µl (approximately 300 cells/embryo). Successfully injected embryos were selected two hours post-injection (dpi). All larvae were incubated at 31 °C for three days (72 h). According to Directive 2010/63/EU on protecting animals used for scientific purposes, zebrafish are considered self-feeding larval forms up to 120 h post-fertilization (hpf), meaning for the first five days of life, and are therefore not classified as animals; thus, ethical approval from the authorities is not required. At the end of the observation period, images of the fish from each group were captured to monitor tumor cell growth. A Discovery V8 Stereo optical microscope and Zeiss hardware were used for the observations. ImageJ software was utilized to calculate the area of tumor cell growth.

### BaseScope™ assay

The in situ hybridization BaseScope^™^ 2.5 Chromogenic Assay (Advanced Cell Diagnostics) was performed according to the manufacturer’s protocol. The cells were seeded in 8-well Nunc Lab-Tek II Chambered Coverglass chambers (Thermo Fisher Scientific). After 24 h, upon reaching 70–80% confluency, the cell medium and the chambers were removed, and the cells were fixed in 10% Neutral Buffered Formalin (Sigma-Aldrich) for 30 min at room temperature. Following three washes in 1 X PBS, the cells were dehydrated using 50, 70, and 100% EtOH for 5 min. The cells were then left in 100% EtOH for an additional 10 min and stored in 100% EtOH at − 20 °C until ready for use. On the day of the assay, the cells were rehydrated by incubating with 70% EtOH for 2 min, 50% EtOH for 2 min, and finally with 1 X PBS for 10 min. A hydrophobic barrier was created between distinct samples using an Immedge hydrophobic barrier pen. Next, the cells were treated with Hydrogen Peroxide for 10 min at room temperature. The slides were washed in 1 X PBS and treated with diluted Protease III (1:15 in 1 X PBS) for 10 min at room temperature. The slides were washed in PBS. From this point forward, all incubations were performed in a wet chamber and oven (steps involving incubation at 40 °C). Target probes (with used probes and their targets listed in Table S2), along with the positive (PPIB) and negative (dapB) control probes, were added to designated samples and incubated for 2 h at 40 °C. After that time, the cells were washed for 2 min in 1 X Wash Buffer two times, and the first hybridization began by adding AMP 1 to completely cover the section, followed by incubation of the slides for 30 min at 40 °C. The cells were washed and incubated with AMP 2 for 30 min at 40 °C, AMP 3 for 15 min at 40 °C, AMP 4 for 30 min at 40 °C, AMP 5 for 30 min at 40 °C, AMP 6 for 15 min at 40 °C, AMP 7 for 30 min at room temperature, and AMP 8 for 15 min at room temperature. Two washes separated each of the steps mentioned above using 1 X Wash Buffer for 2 min. To detect the signal, the Red B solution was diluted in the Red A solution at a 1:60 ratio, immediately added to the slides, and incubated for 10 min at room temperature in darkness. After this step, the slides were washed with water, counterstained with 50% Hematoxylin for 2 min at room temperature, washed three times in water, and submerged in a 0.02% ammonia water solution. Following 10–15 s of incubation, the slides were washed with water approximately three times, and then dried in a hybridization oven for 15 min at 60 °C, after which they were mounted using VectaMount Permanent mounting medium (Vector Laboratories). The slides were air-dried and analyzed with a Leica Stellaris 8 Confocal Laser Scanning Microscope and Leica Application Suite X (LAS X) since the FastRed dye also provides fluorescence. Cell nuclei stained with Hematoxylin were visualized by transmitted light.

### Clonings and transfections

Cells were transfected with plasmids, the generation of which is detailed in Table S3. pLVX-IRES-tdTomato-FlagAkt1 plasmid [67] served as a backbone plasmid to produce plasmids encoding GSN-A, -B, and -C. The RNA isolated from the tested cells using the GenElute^™^ Mammalian Total RNA Miniprep Kit (Sigma-Aldrich) served as a template for the reverse transcription reaction conducted with the High-capacity cDNA reverse transcription kit with RNase inhibitor (Thermo Fisher Scientific). DNA Phusion Hot Start II High-Fidelity Polymerase (Thermo Fisher Scientific) was used to amplify a cDNA fragment. Linearized plasmid and inserts were separated via polyacrylamide gel electrophoresis. All cloning procedures were performed with the NEBuilder HiFi DNA Assembly Cloning Kit (New England BioLabs Inc.).

For stable transfection, cells with GSN knock-out growing in a 35 mm plate were transfected with the appropriate plasmid: pLVX-CMV-zeo, pLVX-CMV-zeo-GSN-A, pLVX-CMV-zeo-GSN-B, or pLVX-CMV-zeo-GSN-C, which code for GSN-A, -B, or -C, respectively, under the CMV promoter and carrying zeocin resistance, using Lipofectamine 3000 (Thermo Fisher Scientific) according to the manufacturer’s recommendations. After deriving the stable lines using the protocol described elsewhere [56], they were verified through immunocytochemistry and Western blot analyses to confirm GSN production. The stable A375 cell clones were cultured in a medium containing 1 µg/ml puromycin from Santa Cruz Biotechnology Inc. and 50 μg/ml zeocin from Thermo Fisher Scientific.

### Coating the plates and dishes with laminin-1

According to the manufacturer's instructions, a laminin-1 (Sigma-Aldrich) solution in Hanks’ Balanced Salt Solution (HBSS) (Thermo Fisher Scientific) at a concentration of 1 µg/cm^2^ was used to coat the coverslip or the cell culture dish. The plates and coverslips with the coating solution were incubated for at least 4 h at 37 °C in a humidified atmosphere containing 5% CO₂. Before seeding the cells, the coated surface was washed three times with PBS.

### Colony formation assay

Five hundred cells per clone were seeded into a well of a 6-well plate containing complete medium. Seven days later, the cells were washed twice with ice-cold PBS and fixed and stained using a staining solution (0.1% crystal violet, 25% methanol in PBS) for 25 min in the dark at room temperature. Next, the cells were washed again with ice-cold PBS to remove any excess stain. Images of the cell colonies were captured, followed by incubation with 10% acetic acid to destain the cells until they became transparent. The absorbance of the dissolved crystal violet was measured at 550 nm using a plate reader.

### Collection of conditioned media

Conditioned medium was collected from a cell culture grown in a 25 cm^2^ culture flask. Cells cultivated in complete medium were washed three times with warm, sterile PBS after reaching approximately 80% confluence and were subsequently cultured in serum-free medium. Conditioned medium was collected after 24 h. To eliminate larger cellular fragments (e.g., apoptotic bodies) and dead cells, the conditioned medium was centrifuged at 4 °C for 20 min at 7000 × *g*. The collected medium (4.5 ml) was filtered through a 200 nm membrane and then concentrated to approximately 0.5 ml using Amicon Ultra-15 Centrifugal Filter Units with a 10 kDa cutoff through centrifugation at 4 °C at 4000 × *g*.

### XTT assay

To perform the XTT assay for assessing mitochondrial metabolic activity, 2000 cells were seeded into a well of a 96-well plate and incubated for 72 h. The assay was then conducted using the CyQUANT™ XTT Cell Viability Assay Kit, following the manufacturer's instructions. The XTT reagent includes tetrazolium salts that are sensitive to the cells' oxidation–reduction potential. These salts are cleaved into formazan through a complex cellular mechanism, which directly correlates with the number of metabolically active cells in the culture. Sample absorbance was measured at 450 and 630 nm using a µQuant plate reader.

### Invasion assay

Transwell^™^ filters (BD Bioscience) were incubated for 24 h in PBS buffer in an atmosphere of 5% CO_2_ at 37 °C. A 1 mg/ml solution of Matrigel^™^ was then prepared in a serum-free medium and added in a volume of 100 µl to each insert. To polymerize the Matrigel^™^, the inserts were incubated for 1 h in an atmosphere of 5% CO_2_ at 37 °C. Cells were cultured in a serum-free medium for 24 h and then subjected to an invasion assay. Cells were trypsinized and counted using a Bürker chamber, and 100,000 cells were plated in serum-free medium in a Transwell^™^ filter onto a layer of Matrigel^™^. A 20% (v/v) FBS solution was used as a chemoattractant in a well of a 24-well plate. After 24 h of incubation in 5% CO_2_ at 37 °C, the cells and the Matrigel^™^ layer were carefully removed from the inner part of the Transwell^™^ filter. The cells that had passed through the Matrigel^™^ layer to the underside of the insert membrane were fixed with a 4% (w/v) formaldehyde/PBS solution and stained with Hoechst 33342, and their nuclei were counted using a FluoView 500 confocal microscope. The experiment was repeated six times for each clone.

### Immunocytochemistry (ICC)

For immunocytochemical staining, cells were seeded on sterile coverslips placed in the wells of a 24-well plate. Forty-five thousand cells were seeded for 24 h, and thirty thousand for 48 h. Samples for TIRF microscopy were prepared by seeding fifteen thousand cells per well in a Nunc^™^ Lab-Tek^™^ Chambered Coverglass (Thermo Fisher Scientific) and incubating the cells for 48 h. Cell fixation was performed by incubating for 20 min at room temperature in a 4% (w/v) formaldehyde/PBS solution. The following cells were incubated in PBS containing 0.1% (v/v) Triton X-100 at room temperature for 6 min and then blocked with 1% (w/v) BSA in PBS at room temperature for 30 min. The coverslips were incubated overnight at 4 °C with a solution of primary antibodies diluted in a blocking solution. Next, the slides were washed three times for five minutes each in PBS and then were incubated for one hour at room temperature in a solution of secondary antibodies diluted in 1% (w/v) BSA in PBS buffer. The cells were washed three times in PBS and once in deionized water and mounted on a microscope slide using Dako mounting medium (Agilent). The antibodies and fluorescent dyes used are listed in Table S1.

### Confocal imaging

Images were taken using a TCS SP8 (Leica) or Stellaris 8 (Leica) confocal laser scanning microscope unless otherwise stated. Typically, all photos were captured with the HC PL APO CS2 63x/1.4 oil objective, and fluorescence detection ranges were configured to capture signals from specific dyes. The detection ranges did not overlap, and the “switch between lines” mode was generally employed for scanning. The pixel size was set to 0.059 μm. Optimal excitation laser wavelengths for specific dyes were adjusted in the case of Stellaris 8 (equipped with a tunable White Laser), while for SP8 imaging, we used fixed wavelength laser lines. Some images (Fig. 2B) taken with Stellaris 8 were acquired using the Lightning module with the “Lightening Grade” parameter set to maximum resolution, “Strategy” set to “Adaptive,” and “Refractive Index” set according to the RI of the mounting medium. Leica Application Suite X (LasX) was utilized to acquire images. The antibodies and fluorescent dyes used are listed in Table S1.

### F:G actin ratio assay

Thirty thousand cells were plated on either non-coated or laminin-1-coated slides within a 24-well plate. After 48 h, the cells were fixed with 4% (w/v) formaldehyde/PBS. The cells were subsequently stained with phalloidin conjugated to the fluorescent dye Alexa Fluor^®^ 488, which binds to F-actin, and with DNase I conjugated to the fluorescent dye Alexa Fluor^®^ 594, which binds to G-actin. Images were captured using a TCS SP8 (Leica) confocal laser microscope in “z-stack” mode, scanning the cells in layers. The F-actin to G-actin ratio was analyzed using the Fiji program. The Z-Projections plug-in was utilized to sum the signal intensity for F- and G-actin separately from all layers of the cell image. Next, the Ratio Plus plug-in was employed to visualize the F:G actin intensity ratio. In the following step, the sum of the pixel values of the image was measured, representing the F-actin to G-actin ratio in the cell. Lookup tables and Rainbow RGB were used to display the cell's F:G actin ratio in a color gradient. Ten cells per cell clone were analyzed, resulting in a total of 30 analyzed cells for each clone group. The data are presented as the mean of the F-actin to G-actin ratio. The analysis was conducted based on a modified protocol described elsewhere [68].

### Gelatin digestion assay

The procedure was performed as described elsewhere [46]. Poly-L-lysine-coated coverslips placed in a 24-well plate were washed with PBS and treated with 0.5% (v/v) glutaraldehyde solution for 15 min at room temperature. The slides were then washed with PBS and incubated for 10 min in a drop of 0.2% (v/v) gelatin-fluorescein solution in PBS. Subsequently, the slides were incubated for 1 min in a 5 mg/ml sodium borohydride solution in PBS before being washed with PBS. Next, 30,000 cells were seeded on each slide coated with the gelatin-fluorescein solution and cultured for 12 h in a complete culture medium at 37 °C in a 5% CO_2_ atmosphere. The cells were then fixed with a 4% (w/v) formaldehyde/PBS solution for 20 min at room temperature. In the next step, F-actin was stained with phalloidin conjugated to Alexa Fluor^®^ 568 dye, while cell nuclei were stained with Hoechst 33342 dye. Images were captured using a TCS SP8 (Leica) confocal laser microscope with Leica Application Suite X (LAS X) software. The gelatin-etched area per single cell was analyzed using Fiji software, with thirty cells per clone group analyzed, and the result was presented as an average.

### Invadopodia number assessment

The Fiji program was utilized to analyze the number and area of invadopodia per cell, employing the threshold cut and analyze particles tools to project their area. For each cell clone, ten cells were examined, resulting in thirty total cells analyzed for each clone group. Data are presented as the average number of invadopodia per cell and their average area projection.

### Filopodia length analysis

For filopodia analysis, cells were seeded onto coverslips that were either uncoated or coated with laminin 1. After 48 h, the cells were fixed and stained with phalloidin-Alexa Fluor^®^ 568. Photographs of the stained cells were taken using a Zeiss Elyra 7 with Lattice SIM and Zen Black software (Zeiss, Jena, Germany) in TIRF mode. Data analysis was conducted with ImageJ software (ImageJ, F. Cordelieres, Institute Curie, Paris, France), which enabled the measurement of the lengths of filopodia cell protrusions. Twenty to thirty cells per group were analyzed. Data are presented as the mean length of the longest filopodium per cell.

### TIRF microscopy

TIRF imaging was conducted using an Elyra 7 Lattice SIM microscope equipped with a 63 × Alpha Plan-Apochromat NA1.46 oil immersion objective. ZEN Black software (version 3.0 SR) was utilized to capture images. The samples were illuminated with laser beams at 488 nm and 568 nm, and beam splitters BP 495–590 and LP 570 were employed. A PCO Edge 4.2 M sCMOS camera collected the fluorescence signal. Processing included merging channels, adjusting brightness (limited to linear changes), applying LUT, and adding scale bars and annotations (e.g., arrows). The antibodies and fluorescent dyes used are detailed in Table S1.

### Projected cell area analysis

The projected cell area was determined using images of cells with fluorescently stained F-actin obtained through TIRF microscopy. These images were analyzed in FIJI (1.54f) using a custom-written script. The main steps involved linear contrast enhancement and filtering with a Gaussian Blur filter, with a sigma parameter set to 10 pixels. This latter step improved cell detection by blurring the edges of the filamentous structures. The FIJI plug-in Cellpose detected cells [69] with the employed model “cyto3” and the diameter set to 30 or 0. The subsequent data were processed in R (version 4.3.1). Objects smaller than 300 µm^2^ were excluded from the analysis. A total of 108 cells were analyzed for the laminin 1 coating condition, while 106 cells were analyzed for the non-coating condition.

### High-content screening system (HCS) to assess the level of adhesion proteins and the spreading of the cells

Cells were cultured and stained in PhenoPlate 96-well, black, optically clear, flat-bottom plates (Revvity). After fixation with 4% (w/v) formaldehyde/PBS, permeabilization with 0.1% (v/v) Triton X-100 in PBS, and blocking with 1% (w/v) BSA in 0.1% (v/v) Triton X-100/PBS, the cells were stained with a combination of anti-integrin β1 or pFAK397 IgG/DAPI/Deep Red Cell Mask and imaged using the Opera Phenix Plus (Revvity) with a 63 × water immersion objective with NA 1.15 (Zeiss). DAPI was excited with a 405 nm laser line, donkey anti-mouse/rabbit-Alexa Fluor™ IgG with a 488 nm laser line, and Cell Mask Deep Red with a 633 nm laser line. Antibodies, as well as fluorescent dyes and their dilutions, are listed in Table S1. Harmony software (version 5.1, Revvity) was used to build a sequence for image analysis blocks to assess the projected cell area and accumulation of β1 integrin at the plasma membrane. The analysis focused on three main blocks: “Find Nuclei,” “Find Cytoplasm,” and “Select Cell Region.” The last block separated the membranous region by expanding the outer cell border (defined by mask staining) by 30% and contracting the outer cell border toward the cell`s interior by 2% (Fig. S10A-A’). All cells touching the image border were removed from the analysis to focus only on the cells present in the image area. Cell Mask Deep Red was used to delineate cells.

pFAK-rich spots were counted in Harmony software (version 5.1). Briefly, maximum intensity projection from the three upper slices was performed for each field of view. The membrane region was defined using the “Select Cell Region” building block, with the outer border set at 20% and the inner border set at 13% (Fig. S10B-B’). The background was removed to enhance FA detection accuracy by subtracting the Gaussian smoothed pFAK channel (with filter width set to 17 pixels) from the original pFAK channel. The resulting images entered the “Find Spots” building block with the selected detection method “D,” Detection Sensitivity set to 0.11, and Splitting Sensitivity set to 0.5.

### Cell migration assays

The migration tests were performed using the IncuCyte Live-Cell Analysis Imaging System (Sartorius) and the manufacturer's dedicated IncuCyte ImageLock 96-well plates, which enable precise time-lapse imaging at specific locations on the plate. For the spontaneous migration test, 1000 cells were seeded in each well and incubated for 72 h in the IncuCyte Live Cell Analysis Imaging System, capturing images every 2 h. The collected images were analyzed using the ManualTracking plug-in (ImageJ). Three parameters describing the properties of migrating cells were determined: the trajectory of cell movement, the distance covered by the cells, and the directionality of movement. The directionality of movement was calculated as the ratio of the cell's straight-line movement (distance to the starting point) to the total distance covered by the cell [70]. Thirty cells were analyzed for each cell clone. To perform the collective migration test, 120,000 cells were seeded into a well coated with laminin and 40,000 cells into a non-coated well. This was done due to the differing surface areas covered by cells depending on the condition. According to our observations, the contact area of cells growing on laminin-1 with the substrate is significantly smaller than that of cells on a non-coated surface [23]. After 24 h, when the cells reached approximately 100% confluence, a scratch was made on the cell monolayer using a Wound Maker tool (Sartorius). The slides were then transferred to the IncuCyte Live Cell Analysis Imaging System and incubated for 72 h, with images captured every 2 h. Migration analysis was performed using IncuCyte software, and data were presented as a percentage of the scratch covered by cells over time. Six images were analyzed for each cell clone.

### Adhesion assay

Cells were seeded in a serum-free medium supplemented with 0.5% BSA, 2 mM CaCl_2_, and 2 mM MgCl_2_ at a density of 35,000 cells per well in a 96-well plate, using either an uncoated or a laminin 1-coated substrate. After 1 h of incubation in a 5% CO_2_ atmosphere at 37 °C, non-adherent cells were removed by washing five times with PBS buffer supplemented with 2 mM CaCl_2_ and 2 mM MgCl_2_. Subsequently, the XTT assay was performed as described above.

### Western blot (WB)

For Western blotting analysis of cell lysates, cells were seeded into a well of a 6-well plate and cultured for 24 h at 37 °C in an atmosphere of 5% CO_2_. Then, the cells were washed three times with cold PBS buffer and scraped in the presence of 300 µl of urea lysis buffer (50 mM Tris–HCl, pH 7.5, 8.6% (w/v) SDS, 8.6% (w/v) sucrose, 74 mM urea, 1 mM DTT, 1:100 serine phosphatase inhibitors, 1:100 tyrosine phosphatase inhibitors, and 1:100 protease inhibitor cocktail). The cell lysate was centrifuged at 12,000 × *g* for 5 min at 4 °C. The supernatant was saved and constituted the test sample. According to the manufacturer's instructions, the protein concentration was measured with the Pierce BCA Protein Assay Kit (Thermo Fisher Scientific) or the Bradford protein assay (Sigma Aldrich). SDS-PAGE was used to separate samples in a 12.5% (v/v) polyacrylamide gel. A semi-wet transfer method was then employed to transfer the separated proteins to a nitrocellulose membrane. Ponceau S nitrocellulose membrane staining was used to control protein loading and transfer efficiency. The membranes were blocked using 5% skimmed milk dissolved in TBS-T buffer (20 mM Tris, 150 mM NaCl, 0.1% (w/v) Tween 20 detergent). Primary and secondary antibodies were diluted in a blocking solution, and their dilutions are listed in Table S1. They were applied to the nitrocellulose membranes and incubated overnight at 4 °C. Unbound antibodies were removed by washing three times with TBS-T buffer. Incubation with HRP-conjugated secondary antibodies diluted in the blocking solution was performed for 1 h, followed by rinsing three times with TBS-T buffer. Protein detection was performed using Clarity Western ECL substrate and the ChemiDoc^™^MP system. Immunoblots were analyzed using Image Lab software.

### Statistical analysis

Analyses were conducted on the data obtained from three OE GSN-CTRL, three OE GSN-A, three OE GSN-B, and three OE GSN-C clones in each experiment, serving as biological replicates. Both graphs and statistical analyses were carried out using GraphPad Prism 7 and 8 (GraphPad Software Inc.). Data presented in the graphs were shown as a mean ± SD. Outliers were identified as values that were two standard deviations below and above the mean. The first step in the statistical analysis involved checking the normality of the data distribution using the Shapiro–Wilk normality test. Further assessments of statistical significance were performed using two-way ANOVA with Dunnett's post hoc multiple comparisons tests. Significance levels were established at *p* < 0.05 (*), *p* < 0.01 (**), *p* < 0.001 (***), and *p* < 0.0001 (****).

## Supplementary Information

Supplementary Material 1

## Data Availability

No datasets were generated or analysed during the current study.

## Abbreviations

ABP: Actin-binding protein
FA: Focal adhesion
F-actin: Filamentous actin
FAK: Focal adhesion kinase
G-actin: Globular actin
GSN: gelsolin
*GSN*: GSN gene
GSN-A: GSN isoform A
GSN-B: GSN isoform B
GSN-C: GSN isoform C
HCS: High-content screening
OE CTRL: Control cells
OE GSN-A: Cells producing GSN-A isoform
OE GSN-B: Cells producing GSN-B isoform
OE GSN-C: Cells producing GSN-C isoform
