# Supplementary material for: Skin melanoma cells produce diverse gelsolin (GSN) isoforms, which play non-redundant roles in cells’ proliferation and motility
Source: Cancer Cell Int. 2025 Jul 1;25:239. doi: 10.1186/s12935-025-03876-x (PMC12211294; doi:10.1186/s12935-025-03876-x)
Supplement: Supplementary file 1 — Supplementary Material 1 [file 12935_2025_3876_MOESM1_ESM.pdf]

SUPPLEMENTARY MATERIAL

Skin melanoma cells produce diverse gelsolin (GSN) isoforms which play non-redundant roles in cells' proliferation and motility

Ewa Mazurkiewicz-Stanek[1¶*], Aleksandra Makowiecka[1], Iryna Kopernyk[1], Michał Majkowski[2], Anna Boguszewska-Czubara[3], Tomasz Trombik[3], Paweł Karpiński[4], Piotr Donizy[5], Antonina J. Mazur[1¶*]

[1]Department of Cell Pathology, Faculty of Biotechnology, University of Wroclaw, Poland, Wrocław, Poland

[2]Faculty of Biotechnology, University of Wroclaw, Wrocław, Poland

[3]Department of Biochemistry and Molecular Biology, Medical University of Lublin, Lublin, Poland

[4]Department of Genetics, Wroclaw Medical University, Wrocław, Poland

[5]Department of Clinical and Experimental Pathology, Wroclaw Medical University, Wrocław, Poland

*Corresponding authors

Email: ewa.mazurkiewicz@uwr.edu.pl (EM-S), antonina.mazur@uwr.edu.pl (AJM)

[¶]These authors contributed equally to this work

Ewa Mazurkiewicz-Stanek, Antonina Joanna Mazur

```
GSN A MAPHRPAPALLCALSLALCALSLPVRAATASRGASQAGAPQGRVPEARPNSMVVEHPEFL
GSN B ---------------------------------------------------MVVEHPEFL
GSN C ------------------------------------------MKLFCCFNSMVVEHPEFL
                                                         *********

GSN A KAGKEPGLQIWRVEKFDLVPVPTNLYGDFFTGDAYVILKTVQLRNGNLQYDLHYWLGNEC
GSN B KAGKEPGLQIWRVEKFDLVPVPTNLYGDFFTGDAYVILKTVQLRNGNLQYDLHYWLGNEC
GSN C KAGKEPGLQIWRVEKFDLVPVPTNLYGDFFTGDAYVILKTVQLRNGNLQYDLHYWLGNEC
      ************************************************************

GSN A SQDESGAAAIFTVQLDDYLNGRAVQHREVQGFESATFLGYFKSGLKYKKGGVASGFKHVV
GSN B SQDESGAAAIFTVQLDDYLNGRAVQHREVQGFESATFLGYFKSGLKYKKGGVASGFKHVV
GSN C SQDESGAAAIFTVQLDDYLNGRAVQHREVQGFESATFLGYFKSGLKYKKGGVASGFKHVV
      ************************************************************

GSN A PNEVVVQRLFQVKGRRVVRATEVPVSWESFNNGDCFILDLGNNIHQWCGSNSNRYERLKA
GSN B PNEVVVQRLFQVKGRRVVRATEVPVSWESFNNGDCFILDLGNNIHQWCGSNSNRYERLKA
GSN C PNEVVVQRLFQVKGRRVVRATEVPVSWESFNNGDCFILDLGNNIHQWCGSNSNRYERLKA
      ************************************************************

GSN A TQVSKGIRDNERSGRARVHVSEEGTEPEAMLQVLGPKPALPAGTEDTAKEDAANRKLAKL
GSN B TQVSKGIRDNERSGRARVHVSEEGTEPEAMLQVLGPKPALPAGTEDTAKEDAANRKLAKL
GSN C TQVSKGIRDNERSGRARVHVSEEGTEPEAMLQVLGPKPALPAGTEDTAKEDAANRKLAKL
      ************************************************************

GSN A YKVSNGAGTMSVSLVADENPFAQGALKSEDCFILDHGKDGKIFVWKGKQANTEERKAALK
GSN B YKVSNGAGTMSVSLVADENPFAQGALKSEDCFILDHGKDGKIFVWKGKQANTEERKAALK
GSN C YKVSNGAGTMSVSLVADENPFAQGALKSEDCFILDHGKDGKIFVWKGKQANTEERKAALK
      ************************************************************

GSN A TASDFITKMDYPKQTQVSVLPEGGETPLFKQFFKNWRDPDQTDGLGLSYLSSHIANVERV
GSN B TASDFITKMDYPKQTQVSVLPEGGETPLFKQFFKNWRDPDQTDGLGLSYLSSHIANVERV
GSN C TASDFITKMDYPKQTQVSVLPEGGETPLFKQFFKNWRDPDQTDGLGLSYLSSHIANVERV
      ************************************************************

GSN A PFDAATLHTSTAMAAQHGMDDDGTGQKQIWRIEGSNKVPVDPATYGQFYGGDSYIIILYNY
GSN B PFDAATLHTSTAMAAQHGMDDDGTGQKQIWRIEGSNKVPVDPATYGQFYGGDSYIIILYNY
GSN C ************************************************************

GSN A RHGGRQGQIIYNWQGAQSTQDEVAASAILTAQLDEELGGTPVQSRVVQGKEPAHLMSLFG
GSN B RHGGRQGQIIYNWQGAQSTQDEVAASAILTAQLDEELGGTPVQSRVVQGKEPAHLMSLFG
GSN C RHGGRQGQIIYNWQGAQSTQDEVAASAILTAQLDEELGGTPVQSRVVQGKEPAHLMSLFG
      ************************************************************

GSN A GKPMIIYKGGTSREGGQTAPASTRLFQVRANSAGATRAVEVLPKAGALNSNDAFVLKTPS
GSN B GKPMIIYKGGTSREGGQTAPASTRLFQVRANSAGATRAVEVLPKAGALNSNDAFVLKTPS
GSN C GKPMIIYKGGTSREGGQTAPASTRLFQVRANSAGATRAVEVLPKAGALNSNDAFVLKTPS
      ************************************************************

GSN A AAYLWVGTGASEAEKTGAQELLRVLRAQPVQVAEGSEPDGFWEALGGKAAYRTSPRLKDK
GSN B AAYLWVGTGASEAEKTGAQELLRVLRAQPVQVAEGSEPDGFWEALGGKAAYRTSPRLKDK
GSN C AAYLWVGTGASEAEKTGAQELLRVLRAQPVQVAEGSEPDGFWEALGGKAAYRTSPRLKDK
      ************************************************************

GSN A KMDAHPPRLFACSNKIGRFVIEEVPGELMQEDLATDDVMLLDTWDQVFVWVGKDSQEEEK
GSN B KMDAHPPRLFACSNKIGRFVIEEVPGELMQEDLATDDVMLLDTWDQVFVWVGKDSQEEEK
GSN C KMDAHPPRLFACSNKIGRFVIEEVPGELMQEDLATDDVMLLDTWDQVFVWVGKDSQEEEK
      ************************************************************

GSN A TEALTSAKRYIETDPANRDRRTPITVVKQGFEPPSFVGWFLGWDDDYWSVDPLDRAMAELAA
GSN B TEALTSAKRYIETDPANRDRRTPITVVKQGFEPPSFVGWFLGWDDDYWSVDPLDRAMAELAA
GSN C TEALTSAKRYIETDPANRDRRTPITVVKQGFEPPSFVGWFLGWDDDYWSVDPLDRAMAELAA
      **************************************************************
```

Fig. S1. Comparison of amino acid sequences of studied GSN isoforms. Alignment of amino acid sequences of human GSN isoforms analyzed in the study, i.e., A, B, and C. “Asterisk” - no substitutions; “dot” – semi-conserved substitution. The signal peptide of the secretory isoform is shown in red, while M, shown in green, indicates the beginning of the GSN-B isoform. CLUSTAL format alignment was performed using MAFFT (v7.511) (Kuraku et al., 2013; Katoh et al., 2019).

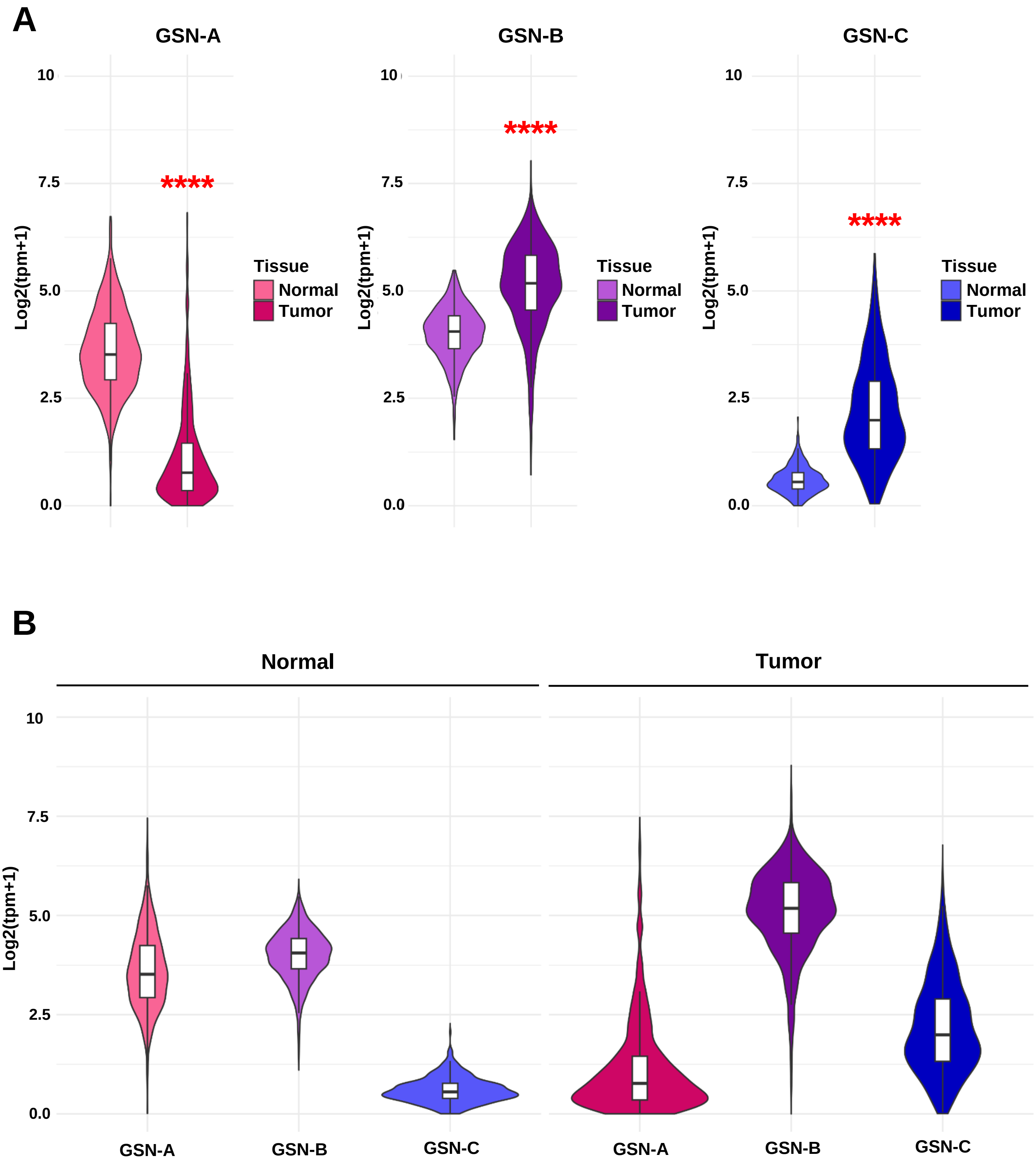

Fig. S2. Transcripts of GSN-A, -B and –C are produced by normal and melanoma skin tissue. (A-B) Analysis of GSN-A, -B, and -C transcript coding levels in GTEX SKIN and TCGS SKCM databases. For normal skin tissue, 555 RNA-seq profiles were analyzed, while for skin melanoma tissue, 469 RNA-seq profiles. TPM levels of GSN isoforms between healthy and tumor tissues were compared using the Kruskal-Wallis test. A *p*-value ≤ 0.05 was considered statistically significant.

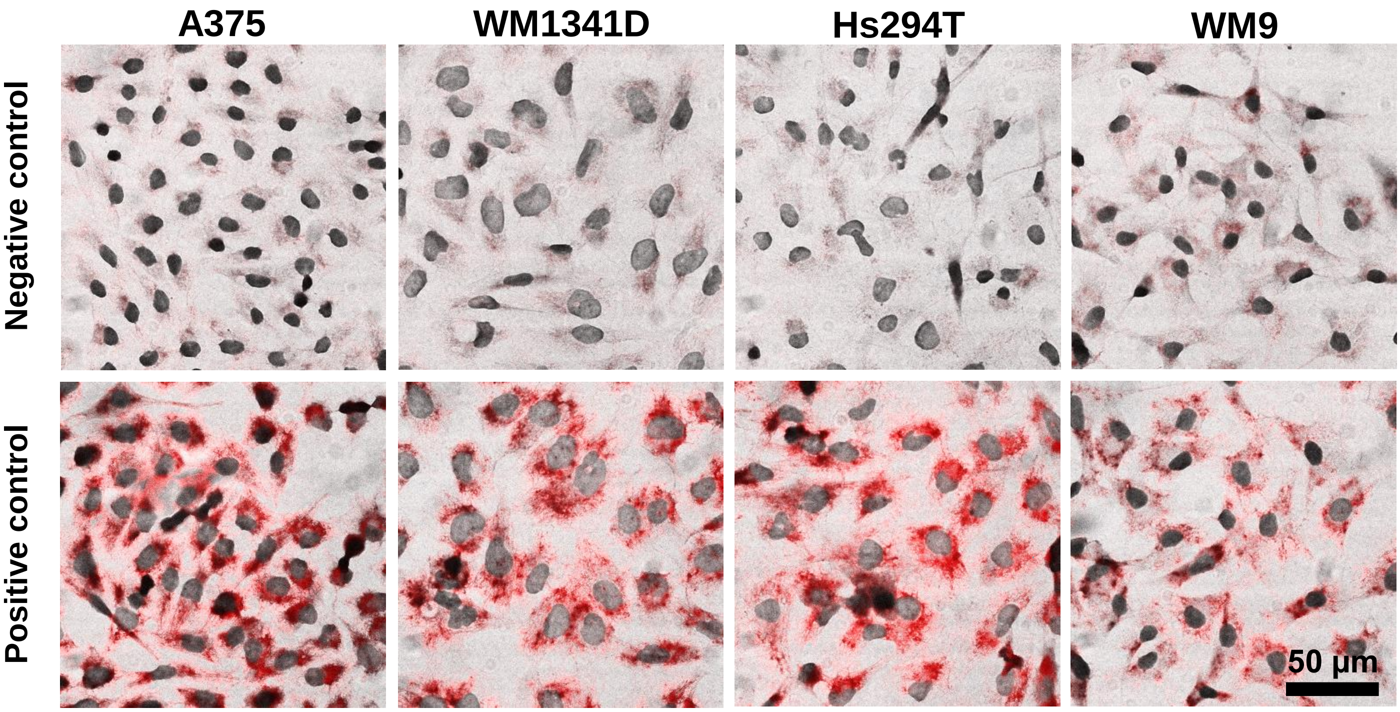

Fig. S3. BaseScope™ analysis of four melanoma cell lines – negative and positive controls. As positive control served a probe detecting mRNA coding for human protein PPIB, while as negative control served a probe targeting mRNA coding for dapB, a protein from *Bacillus subtilis* strain SMY, a soil bacterium.

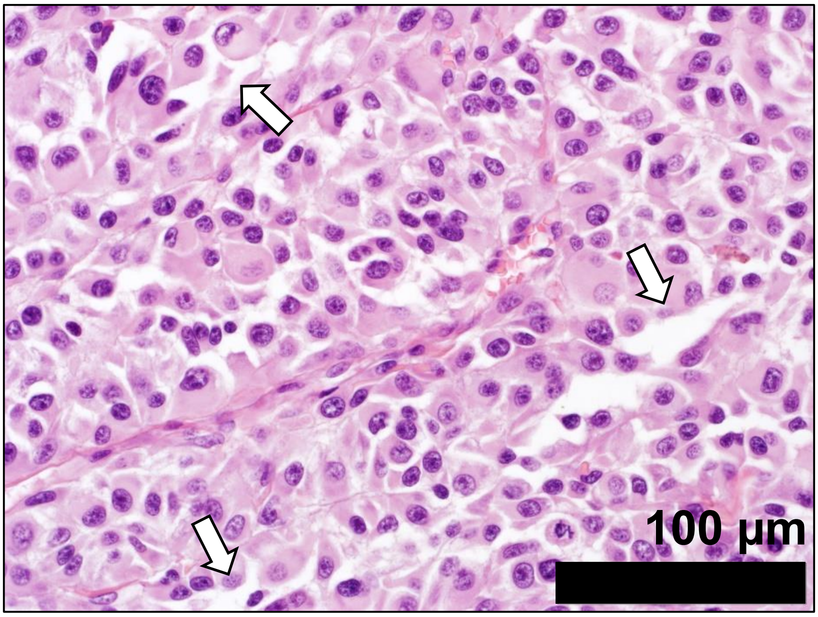

Fig. S4. Detection of presence of extracellular spaces in the tumor area with disrupted cellular adehsion. Human skin melanoma tissue sample was subjected to histopathological staining procedure and stained with eosin (pink) and hematoxylin (blue) to detect cytoplasm and nucleus, respectively. The same melanoma case was analyzed as in Fig. 1H'. White arrows point at tumor areas with disrupted cellulr adhesion.

A

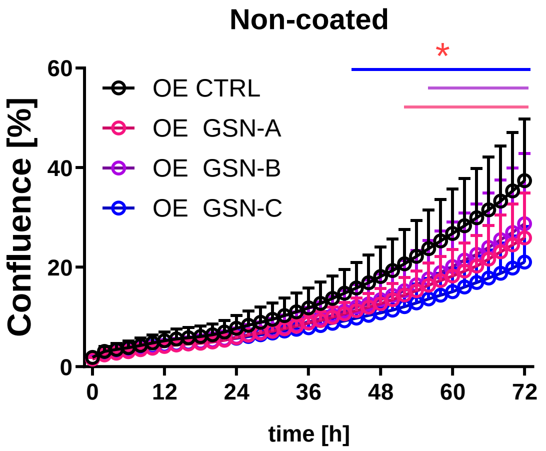

Non-coated

Time [h]	OE CTRL vs. OE GSN-A	OE CTRL vs. OE GSN-B	OE CTRL vs. OE GSN-C
2-42	-	-	-
42-50	-	-	**
53-54	*	-	***
56-60	**	*	****
60-64	***	*	****
66-72	****	**	****

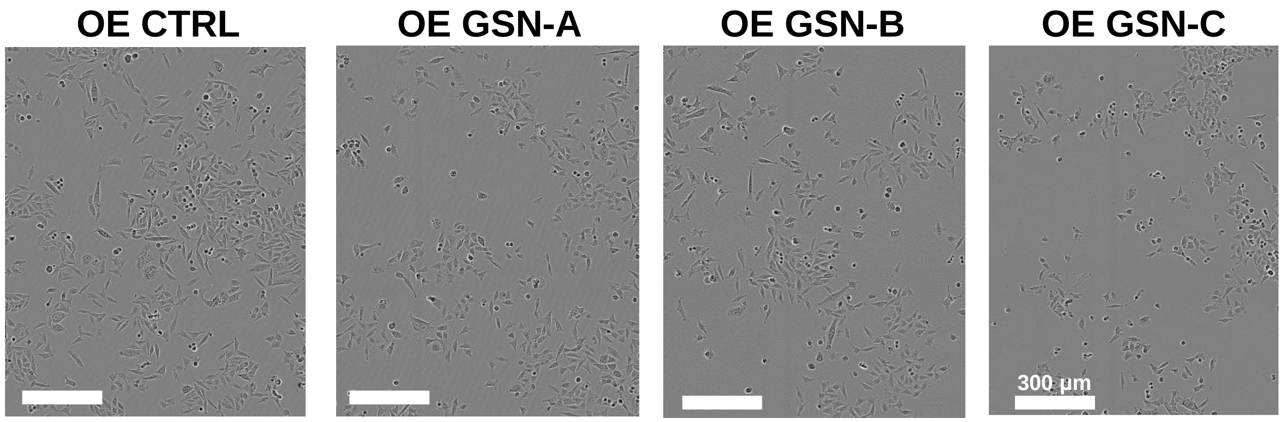

B

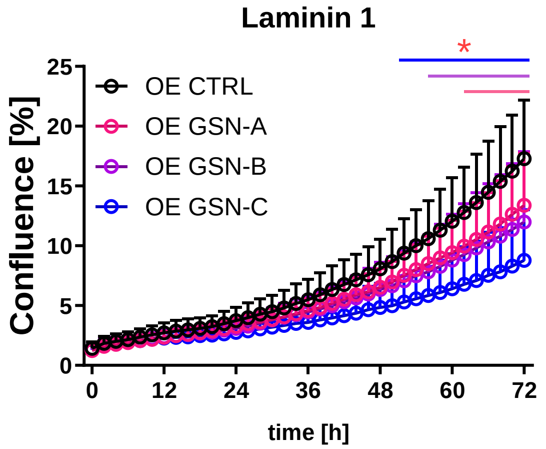

Laminin 1

Time [h]	OE CTRL vs OE GSN-A	OE CTRL vs OE GSN-B	OE CTRL vs OE GSN-C
2-42	-	-	-
44-48	-	-	*
50-54	-	-	***
56	-	*	****
58-60	-	*	****
62	*	**	****
64-66	**	***	****
68-72	**	****	****

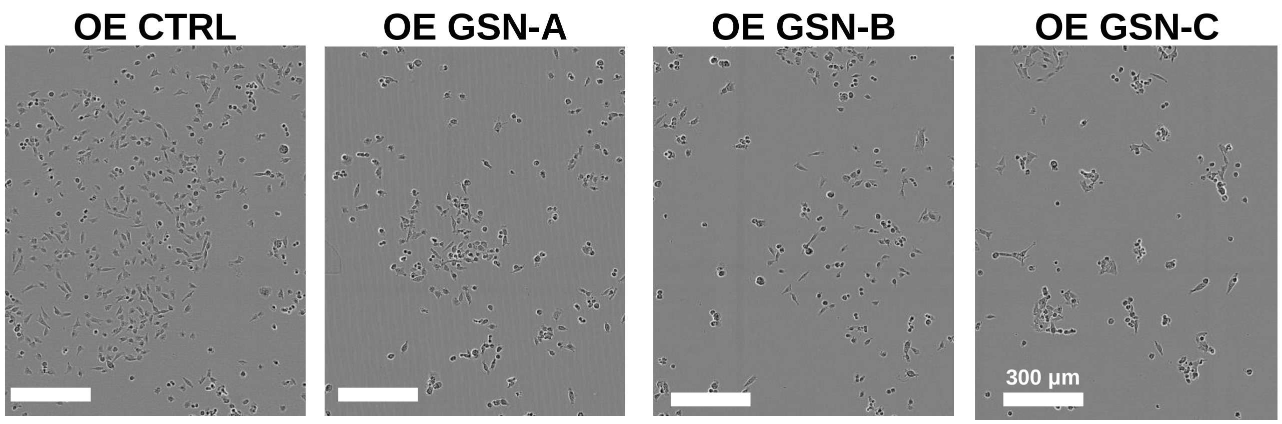

Fig. S5. Evaluation of confluence of cells grown on non-coated (A) or laminin 1-coated (B) surfaces. Analysis performed using the IncuCyte system. Pictures of cells growing in wells of a 96-well plate were taken every two hours for 72 hours. The % confluence of cells was then calculated. Tables showing statistical significance (n=9). Results are presented as mean ± SD; $p \leq 0.05$ (*), $p \leq 0.01$ (**), $p \leq 0.001$ (***), and $p \leq 0.0001$ (****); two-way ANOVA and Dunnett's multiple comparison test.

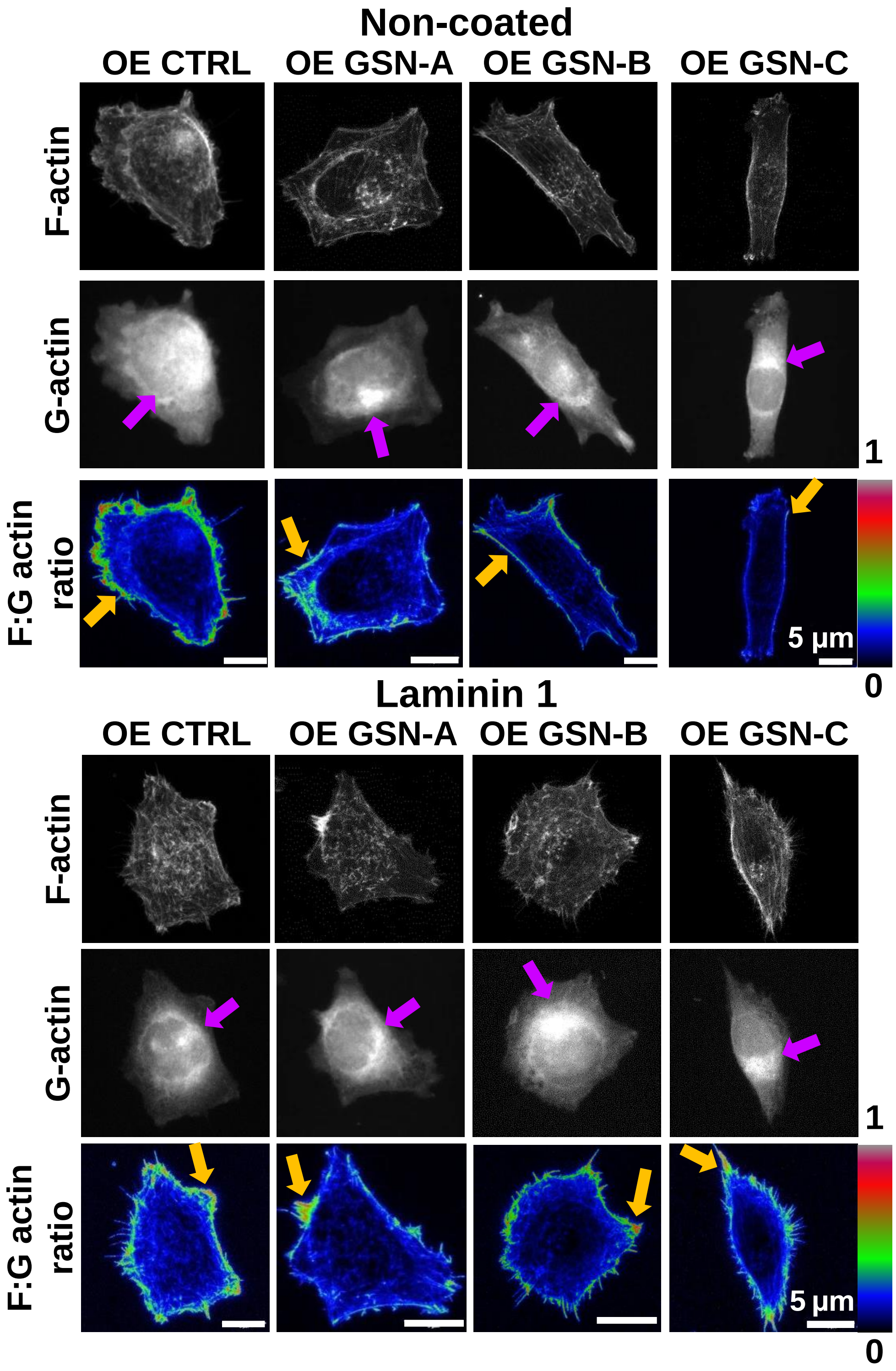

Fig. S6. Actin polymerization state in cells expressing individual gelsolin isoforms. Representative images of cells incubated for 48 h on non-coated or laminin-1-coated coverslips, stained for G-actin and F-actin. Z-axis images of cell layers were taken using a confocal microscope. The Z-axis distribution of the sum of fluorescence intensities for F-actin and G-actin, as well as the ratio of the sum of F-actin to G-actin intensities within the cell, was then visualized using Fiji software. Color coding: black encodes minimum values, red encodes maximum values. Orange arrows indicate the cell periphery, where the highest F:G-actin ratio was observed.

A

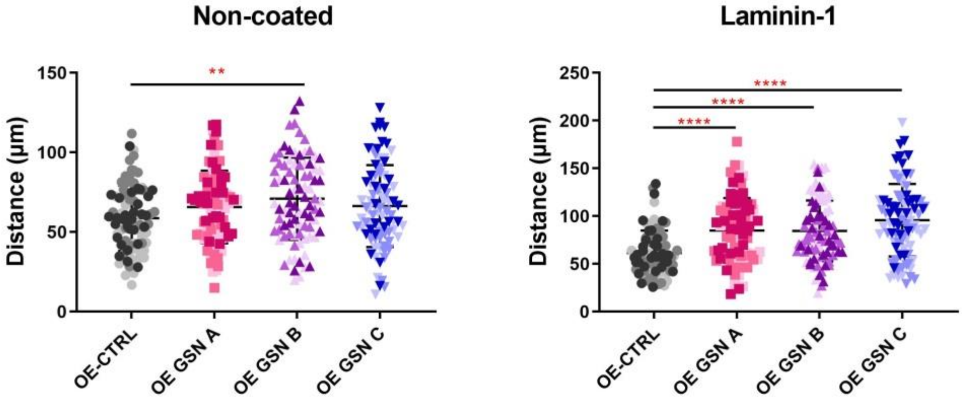

B

Non-coated

Time [h]	OE CTRL vs OE GSN-A	OE CTRL vs OE GSN-B	OE CTRL vs OE GSN-C
2-26	-	-	-
28-32	-	-	-
34-36	-	-	-
38-42	**	-	**
44	**	-	***
48	***	-	***
50-72	***	-	****

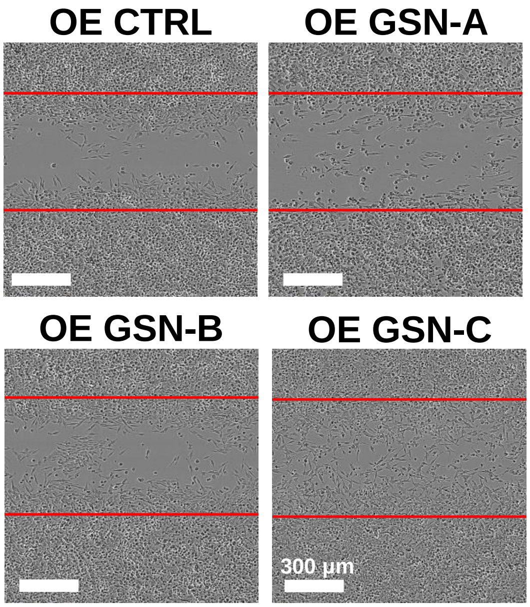

Laminin-1

Time [h]	OE CTRL vs OE GSN-A	OE CTRL vs OE GSN-B	OE CTRL vs OE GSN-C
2-30	-	-	-
32-38	*	-	-
40-42	*	*	-
44-66	**	*	-
60-72	*	*	-

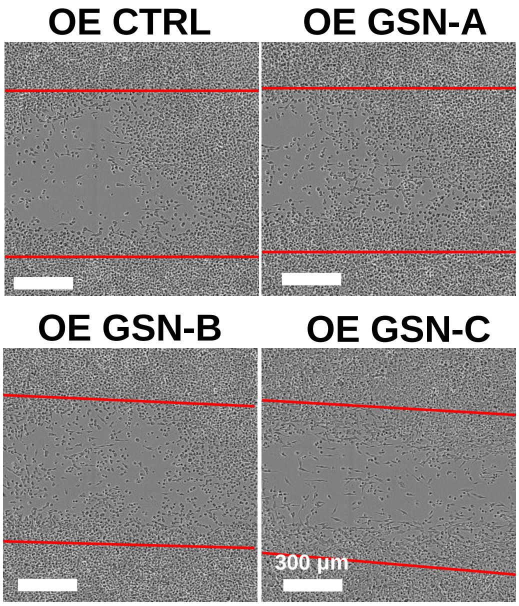

Fig. S7. Migration of cells producing individual gelsolin isoforms growing on non-coated or laminin 1-coated surfaces. Analysis performed using the IncuCyte system. **(A)** Spontaneous migration. The experiment was conducted for 72 h, and the distance traveled (n=90) by migrating cells was then calculated. **(B)** Collective migration. After creating a scratch using the WoundMaker tool, cells were monitored for 72 h, with images taken every two hours. The area of scratch healing by cells was then counted over time. Tables show statistical significance (n=9). Results are presented as mean ± SD; *p* ≤ 0.05 (*), *p* ≤ 0.01 (**), *p* ≤ 0.001 (***), and *p* ≤ 0.0001 (****); two-way ANOVA and Dunnett's multiple comparisons test. Representative photos were taken 72 h after making the scratch.

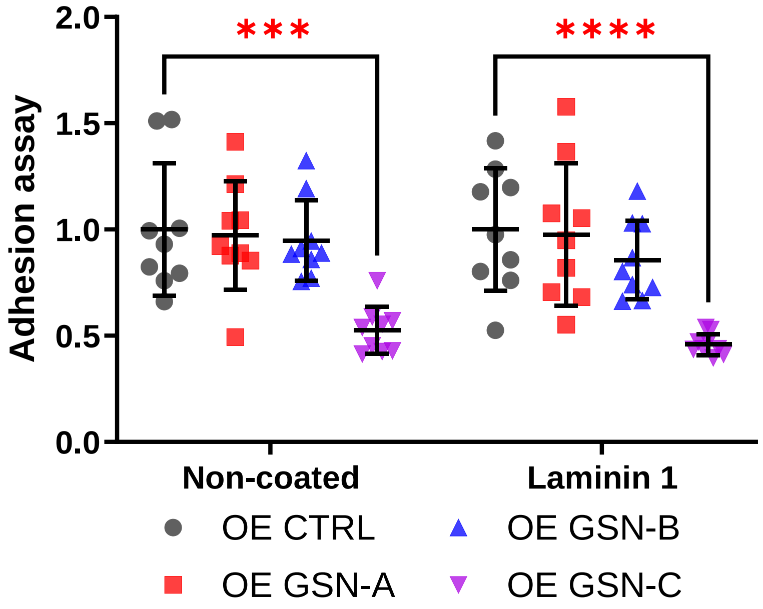

Fig. S8. Adhesion assay based on the XTT assay. Cells were seeded into non-coated or laminin 1-coated wells of a 96-well plate. After two hours, the wells were washed twice with PBS, and the cells were subjected to the XTT assay (n=9). Results are presented as mean ± SD; $p \leq 0.001$ (***), and $p \leq 0.0001$ (****); two-way ANOVA and Dunnett's multiple comparison test.

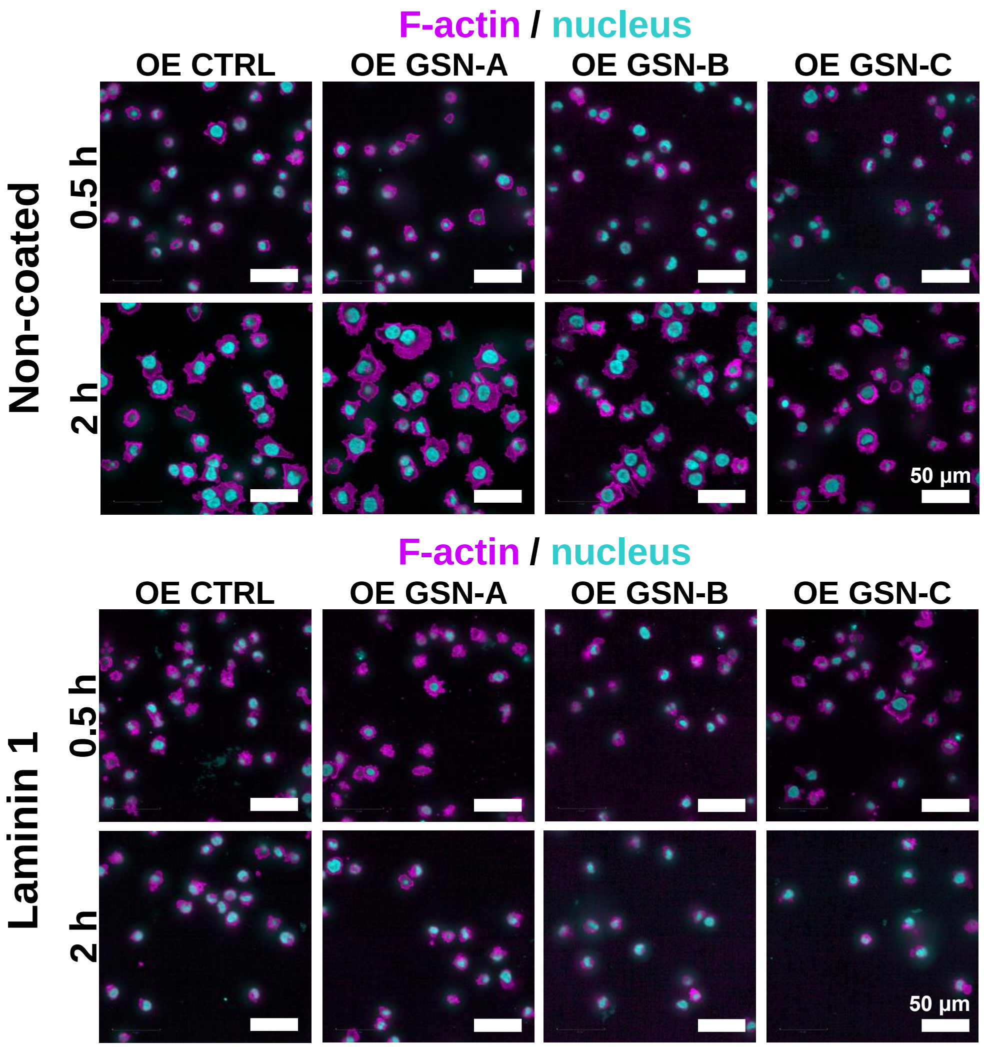

Fig. S9. Spreading ability of the analyzed cells. The cells were seeded into non-coated or laminin 1-coated wells of a PhenoPlate 96-well plate (Revvity). 30 min or 2 hours later, the cells were washed twice with PBS, fixed and stained with fluorescently labelled phalloidin and Hoechst 33342 to detect F-actin and the cell nucleus, respectively. Images were acquired with Opera Phenix Plus (Revvity) - High Content Screening (HCS) system. 53 fields per cell clone were analyzed.

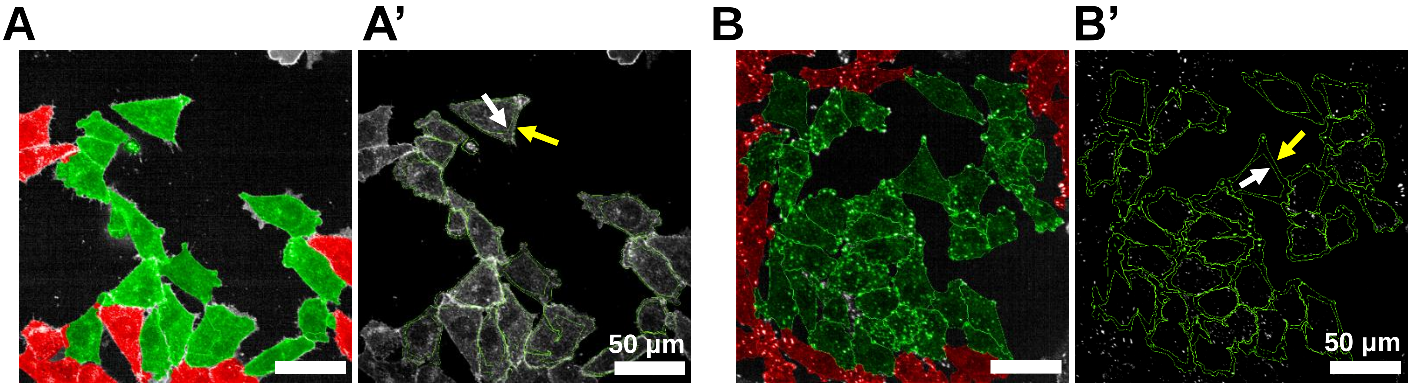

Figure S10. Membranous region determination in Harmony (Revvity) software. To separate the membranous region of the cell staining, HCS CellMask™ Deep Red Stain was used. **(A** and **B)** Entire single field of view. Cells in the red show an image border touching objects excluded from processing and analysis. **(A'** and **B')** The region between the two lines indicates the membranous region in which fluorescence intensity was measured. The membranous region was separated by expanding the outer cell border (defined by mask staining) by 30% (A' integrin β1 detection) or 20% (B' pFAK^{397} detection) (yellow arrow) and contracting the outer cell border toward the cell`s interior by 2% (A' integrin β1 detection) or 13% (B' pFAK^{397} detection) (white arrow).

Table S1. List of antibodies and fluorescent dyes used in the study.

Antibody, catalogue number	Company	IHC	ICC	WB
mouse anti-GSN IgG, G4896	Sigma-Aldrich	-	1:200	1:4000
rabbit anti-GSN IgG, EPR1942	Abcam	1:200	-	-
rabbit anti-Sox10 IgG,	Cell Marque Antibodies	1:100	-	-
mouse anti-β-tubulin, T4026	Sigma-Aldrich	-	-	1:1000
mouse anti-GM130 IgG, 610823	BD Biosciences	-	1:100	-
mouse anti-GALNT2, IgG, AF7507	Bio-techne	-	1:100	-
rabbit anti-$pFAK^{397}$, 8556	Cell Signaling	-	1:200	-
mouse anti-Integrin β1 IgG, CBL481	Merck-Millipore	-	1:150	-
mouse anti-GAPDH IgG, 47724	Santa Cruz Biotechnology Inc.	-	-	1:200
HRP-conjugated anti-rabbit IgG, 7074	Cell Signaling	-	-	1:4000
HRP-conjugated anti-mouse IgG, 7076	Cell Signaling	-	-	1:4000
donkey anti-mouse IgG Alexa Fluor™ 488, A-21202	Invitrogen	-	1:200	-
donkey anti-mouse IgG Alexa Fluor™ 568, A-10037	Invitrogen	-	1:200	-
donkey anti-rabbit IgG Alexa Fluor™ 488, A-21206	Invitrogen	-	1:200	-
donkey anti-rabbit IgG Alexa Fluor™ 568, A-10042	Invitrogen	-	1:200	-

Fluorescent dye, catalogue number	Company	ICC
Hoechst 33342, H21492	Sigma-Aldrich	5 mg/ml
Phalloidin-Alexa Fluor™ 568, A123-80	ThermoFisher Scientific	1:100-1:400
Phalloidin-CruzFluor™ 488, 363791	Santa Cruz Biotechnology Inc.	1:1000
DNase I-Alexa Fluor™ 594, D12371	ThermoFisher Scientific	1:100
HCS CellMask™ Deep Red Stain, H32721	ThermoFisher Scientific	1:20,000

Table S2. Numbers (from Pubmed database) of transcripts coding GSN a, b or c recognized by probes used in BaseScope™ assay.

GSN isoform	Transcript number/Accession no.	Probe name
GSN-A	NM_000177.5	BaseScope Probe BA-Hs-GSN-tv1-E1E2-C1
GSN-B	NM_198252.3	BaseScope Probe BA-Hs-GSN-tv2-E2E3-C1
GSN-C	NM_001353070.2	BaseScope Probe BA-Hs-GSN-tv28-E3E4

Table S3. List of used plasmids in the study and description of their generation.

Plasmid	Primers sequences	Description
pLVX-IRES-CMV-GSN-B-zeo	IRES fwd:<br>5'→gaaggatgcccagaaggtaccccattgtatgggatctgatc<br>IRES rev:<br>5'→acttggccatattatcatcgtgtttttcaaag<br>WPRE fwd:<br>5'→gcaggactgaacgcgtctggaacaatcaac<br>WPRE rev:<br>5'→gtcattggtcttaaaggtacctgaggtgtgactggaaaacc<br>Zeocin fwd:<br>5'→cgatgataatatggccaagttgaccagtg<br>Zeocin rev:<br>5'→ccagacgcgttcagtcctgctcctctgc<br><br>GSN-B fwd:<br>5'→tctactagaggatctatttccggtgaattcaccatggtggtggaacacc<br>GSN-B rev:<br>5'→tagggggggggggagggagaggggcgggatcctcaggcagccagctcagc	pLVX-IRES-tdTomato-FlagAkt1 plasmid (Kajno et al., 2015) was cut with *Kpn*I restriction enzyme to remove the WPRE sequence, part of IRES sequence and sequence coding for tdTomato. IRES and WPRE sequences were reintroduced. In the same Gibson reaction (NEBuilder HiFi DNA Assembly Master Mix), the sequence coding for zeocin resistance was cloned into the plasmid. The nucleotide sequence coding for zeocin was PCR amplified using the pDRIVE5LUCIA-mDesmin (IvivoGen) plasmid as the template. The obtained plasmid was called pLVX-IRES-zeo. Next, *EcoR*I and *BamH*I restriction enzymes were utilized to remove the sequence encoding FlagAkt1 from the pLVX-IRES-zeo plasmid and the nucleotide sequence coding for GSN-B amplified by PCR from the cDNA of the A375 cells was cloned into the cut plasmid.
pLVX-IRES-CMV-GSN-A zeo	GSN-A fwd:<br>5'→tagaggatctatttccggtgaattcgccaccatggctccgcac<br>GSN-A rev:<br>5'→tagggggggggggagggagaggggcgggatcctcaggcagccagctcagc	The nucleotide sequence coding for GSN-A was amplified by PCR from the cDNA of A375 cells and cloned into the pLVX-IRES-CMV-GSN-B-zeo plasmid, which had been cut with *EcoR*I and *BamH*I restriction enzymes to remove the sequence coding for GSN-B.
pLVX-IRES-CMV-GSN-C zeo	GSN-C fwd:<br>5'→tagaggatctatttccggtgaattcaccatggaaaaactgttttgttg<br>GSN-C rev:<br>5'→tagggggggggggagggagaggggcgggatcctcaggcagccagctcagc	The nucleotide sequence coding for GSN-C was amplified by PCR from the cDNA of A375 cells and cloned into the pLVX-IRES-CMV-GSN-B-zeo plasmid, which had been cut with *EcoR*I and *BamH*I restriction enzymes to remove the sequence coding for GSN-B.
pLVX-IRES-CMV-zeo	n.a.	The plasmid pLVX-IRES-CMV-GSN-B-zeo was cut with *EcoR*I and *BamH*I restriction enzymes (removal of the sequence coding for GSN-B). Next, it was treated with T4 polymerase to obtain blunt ends; ligation was performed using T4 DNA ligase.

References:

Kajno, McGraw, Gonzalez, 2015. Development of a new model system to dissect isoform specific Akt signalling in adipocytes. *Biochemical Journal*, DOI: 10.1042/BJ20150191

Katoh, Rozewicki, Yamada, 2019. MAFFT online service: multiple sequence alignment, interactive sequence choice and visualization. *Briefings in Bioinformatics*, DOI: 10.1093/bib/bbx108

Kuraku, Zmasek, Nishimura, Katoh, 2013. aLeaves facilitates on-demand exploration of metazoan gene family trees on MAFFT sequence alignment server with enhanced interactivity. *Nucleic Acids Research*, DOI: 10.1093/nar/gkt389
